# Correction: ‘Facilitating interprofessional learning: experiences of using a digital activity for training handover of critically ill patients between a primary health care centre and ambulance services – a qualitative study’

**DOI:** 10.1136/bmjopen-2023-083585corr1

**Published:** 2024-08-13

 Taloyan M, Helen C, Ninni Å, *et al*. Facilitating interprofessional learning: experiences of using a digital activity for training handover of critically ill patients between a primary health care centre and ambulance services – a qualitative study. BMJ Open 2024;0:e083585. doi:10.1136/ bmjopen-2023-083585

This article has been corrected since it was published online. This article has been corrected since it was published online. The order of the authors has been updated from ‘Conte Helen, Marina Taloyan, Åkesson Ninni, Sofie Guldbrand, Veronica Lindström’ to ‘Marina Taloyan, Conte Helen, Åkesson Ninni, Sofie Guldbrand, Veronica Lindström’.

