# Supplementary material for: Facilitating interprofessional learning: experiences of using a digital activity for training handover of critically ill patients between a primary health care centre and ambulance services – a qualitative study
Source: BMJ Open. 2024 Jun 21;14(6):e083585. doi: 10.1136/bmjopen-2023-083585 (PMC11298706; doi:10.1136/bmjopen-2023-083585)
Supplement: Author's manuscript [file bmjopen-2023-083585.draft_revisions.pdf]

BMJ Open

BMJ Open is committed to open peer review. As part of this commitment we make the peer review history of every article we publish publicly available.

When an article is published we post the peer reviewers' comments and the authors' responses online. We also post the versions of the paper that were used during peer review. These are the versions that the peer review comments apply to.

The versions of the paper that follow are the versions that were submitted during the peer review process. They are not the versions of record or the final published versions. They should not be cited or distributed as the published version of this manuscript.

BMJ Open is an open access journal and the full, final, typeset and author-corrected version of record of the manuscript is available on our site with no access controls, subscription charges or pay-per-view fees (http://bmjopen.bmj.com).

If you have any questions on BMJ Open's open peer review process please email info.bmjopen@bmj.com

BMJ Open

Interprofessional learning in emergency medical and primary health care services using a digital activity– a qualitative study

Journal:	*BMJ Open*
Manuscript ID	bmjopen-2023-083585
Article Type:	Original research
Date Submitted by the Author:	22-Dec-2023
Complete List of Authors:	Taloyan, Marina; Karolinska Institute, Department of Neurobiology, Care Sciences and Society, Division of Family Medicine and Primary Care; Region Stockholm, Academic primary health care center<br>Helen, Conte; Karolinska Institutet, Department of Neurobiology, Care Sciences and Society, Division of Nursing<br>Ninni, Åkesson; Region Stockholm , Rehab North West<br>Guldbrand, Sofie; Region Stockholm, Child Health Care Centre<br>Lindström, Veronica; Umeå University, Department of Nursing and Department of Ambulance Service; Sophiahemmet Hogskola, Department of Health Promotion Science
Keywords:	MEDICAL EDUCATION & TRAINING, ACCIDENT & EMERGENCY MEDICINE, Primary Health Care

SCHOLARONE™
Manuscripts

I, the Submitting Author has the right to grant and does grant on behalf of all authors of the Work (as defined in the below author licence), an exclusive licence and/or a non-exclusive licence for contributions from authors who are: i) UK Crown employees; ii) where BMJ has agreed a CC-BY licence shall apply, and/or iii) in accordance with the terms applicable for US Federal Government officers or employees acting as part of their official duties; on a worldwide, perpetual, irrevocable, royalty-free basis to BMJ Publishing Group Ltd ("BMJ") its licensees and where the relevant Journal is co-owned by BMJ to the co-owners of the Journal, to publish the Work in this journal and any other BMJ products and to exploit all rights, as set out in our licence.

The Submitting Author accepts and understands that any supply made under these terms is made by BMJ to the Submitting Author unless you are acting as an employee on behalf of your employer or a postgraduate student of an affiliated institution which is paying any applicable article publishing charge ("APC") for Open Access articles. Where the Submitting Author wishes to make the Work available on an Open Access basis (and intends to pay the relevant APC), the terms of reuse of such Open Access shall be governed by a Creative Commons licence – details of these licences and which Creative Commons licence will apply to this Work are set out in our licence referred to above.

Other than as permitted in any relevant BMJ Author's Self Archiving Policies, I confirm this Work has not been accepted for publication elsewhere, is not being considered for publication elsewhere and does not duplicate material already published. I confirm all authors consent to publication of this Work and authorise the granting of this licence.

For peer review only

Interprofessional learning in emergency medical and primary health care services using a digital activity– a qualitative study

Authors: Taloyan Marina[1,2*] Conte Helen[3*,] Åkesson Ninni[4], Guldbrand Sofie[5], Lindström Veronica[3,6]

Affiliations:

[1]Academic primary health care center, Region Stockholm, Sweden

[2]Department of Neurobiology, Care Sciences and Society, Division of Family Medicine and Primary Care, Karolinska Institutet, Stockholm, Sweden

[3]Department of Neurobiology, Care Sciences and Society, Section of Nursing, Karolinska Institutet, Stockholm, Sweden

[4] Rehab North West, Region Stockholm, Sweden

[5] Child Health Care Centre, Region Stockholm; Sweden

[6]Department of Nursing, Umeå University & Department of Ambulance Service, Region Västerbotten, Umeå, Sweden.

[*] co-first authorship

Word count: 3419

Corresponding author:
Veronica Lindström
Orcid ID: 0000-0003-1386-3203
Department of Nursing, Umeå University and Division of ambulance service, Region of Västerbotten, Sweden
e-mail: veronica.lindstrom@umu.se
Telephone: +4670-3428157

Abstract

Objective: To explore students' and facilitators' experiences of using a digital IPL activity focusing on acute patient handovers from Primary Health Care to the Emergency Medical Service. **Design**: A qualitative study design was employed. **Method:** Four digital IPL activities were conducted in 2021. Thematic analysis was used to capture the experiences of 31 participants, including 22 students from five different healthcare professions, seven

For peer review only
For peer review only - http://bmjopen.bmj.com/site/about/guidelines.xhtml

facilitators, and two observers. **Setting:** The study was conducted in Primary Health Care to the Emergency Medical Service. The reporting of this study adheres to the COREQ checklist. **Results:** The activity enabled the students to find an interprofessional model for collaboration through reasoning, by communicating and sharing knowledge with the support of a common structure. Participants perceived the digital IPL activity and scenario as authentic, feasible, and supportive for IPL. Using a case with an acute life-threatening condition was a success factor for students to experience high realism in their IPL on patient safety, handover, care, and treatment**. Conclusion:** A digital IPL activity supports students' IPL, allows reaching a higher number of participating students and facilitators, and exhibits potential sustainability. However, a comprehensive evaluation, both quantitative and qualitative, over an extended period of clinical rotations and involving a larger group of students is warranted. **Patient and Public Involvement:** Neither patient nor the public was involved in this study.

Keywords: Feasibility**,** Interprofessional learning, Interprofessional education, Patient safety; Digital learning activity; Chain of care; Emergency medical service; Primary healthcare care service

Strengths and limitations of this study (five bullet points)

- The authors' preconceptions and personal experiences may have influenced the interpretation and understanding of the study.
- Multiple data sources (interviews, notes, observations) and 31 participants creating new knowledge based on qualitative analysis.
- To our knowledge, the first study using data from IPL learning activities with students in clinical rotations in both the Primary Health Care and Emergency Medical services

For peer review only

For peer review only - http://bmjopen.bmj.com/site/about/guidelines.xhtml

- The number of participants and completed IPL activities could be considered low and we do not know if all eligible students were reached.
- Not all professions among the students were representative during all IPL activities.

Introduction

The number of healthcare students doing clinical rotations is increasing and the availability of clinical placements does not match the increased need. This imbalance has necessitated the identification of alternative sites and ways for learning together in pairs or groups. Given the increased number of students, it is essential to establish interprofessional learning (IPL) activities that ensure high-quality clinical education. This is particularly critical in clinical settings where IPL is still in the early stages of development. In the Emergency Medical Services (EMS), interprofessional learning (IPL) is not yet firmly established. Historically, the EMS has not been considered an optimal clinical rotation. However, it is shown that within the EMS, students can gain proficiency in nursing, medical, and collaborative skills [1]. The study findings in addition to the need for clinical placements have contributed to a higher enrolment of students in the EMS and the Primary Healthcare Centre (PHC). Given the growing number of students, there is a need to develop easily accessible IPL activities. Therefore, this study aimed to explore students' and facilitators' experiences of using a digital IPL activity focusing on acute patient handovers from Primary Health Care to the Emergency Medical Service.

Background

IPL includes a range of activities, e.g., short digital seminars or shadowing where the students' learning originates from interactive group processes including collaborative analysis and joint critical reflection [2]. The students are active participants and learn with and from each other in authentic and patient-centred activities [3]. They negotiate their different experiences to

For peer review only - http://bmjopen.bmj.com/site/about/guidelines.xhtml

fully understand their own role and the roles of other professions [4]. However, finding places, spaces, and time for students from different educational programs to learn together in healthcare can be a challenge since education is not always situated in contexts that support IPL [5]. Environments and care activities that naturally could support IPL are situated during clinical rotations where students from different educational programs collaborate in patient care [6, 7]. Students acknowledged that experiencing a diversity of IPL activities during clinical rotations is vital for learning how to work together [8]. Co-location is important but not enough for different professions to learn together [8]. Facilitated learning is part of building interprofessional rapport in groups and balancing the participation of the different professions in their interactions is vital for learning together [1, 4, 8]. Students suggest that bringing a sense of their profession into the interactions and being inclusive of other professions are two key qualities for IPL [8] and that gaps in role conceptions are a crucial barrier [9] and could cause risks for patients.

The World Health Organization (WHO) states that healthcare services around the world should be safe, and person-centred [10]. Research has shown that different IPL activities can introduce patient safety concepts and experiential practice through interprofessional communication and analysis among students [11, 12]. A situation that presents a natural opportunity for IPL is the handover of patients from PHC to the EMS in acute situations. The handover between different healthcare settings are complex process that includes communicating information between professionals responsible for the patient's care at different units or sites [13] and communication failures are one of the main causes of adverse events, particularly in handover situations [14]. When the handover is compromised, essential information can be altered or lost, transfer of cognitive bias may occur, patients may be exposed to adverse events and patient safety issues may occur [15]. To improve patient safety

For peer review only - http://bmjopen.bmj.com/site/about/guidelines.xhtml

in the healthcare setting, training must start with students [16, 17]. Expanding IPL activities beyond organizational boundaries to include the transfer of patients between different healthcare organizations could be a possibility for students to train in handover and patient safety in a new context. The EMS collaborates with PHC and community-based care and is involved in both receiving and handing over patients within these organizations [18, 19]. To our knowledge, the handover of patients from PHC to the EMS in an acute situation has not previously been utilized for IPL.

Methods

Design

This study focuses on students' and facilitators' experiences of using a digital IPL activity. By exploring their experiences new knowledge on IPL in PHC and EMS is created. Additionally, the acceptability of the digital IPL activity designed is an appropriate, relevant, or sustainable learning activity can be explored and described. Acceptability is one main area and captures how the intended recipients react to the intervention [20]. A qualitative design was used for collecting and analysing both the students' and facilitators' experiences and acceptability through reflections on and on the activity. The consolidated criteria for reporting qualitative research COREQ guidelines were used to guide the study [21]. The study was approved by the ethics review authority in Sweden (No:2021-00586).

Patient and Public Involvement

Neither patient nor the public was involved in this study

Setting

The EMS and PHC provide clinical rotations for medical and nursing students. The PHC also provides clinical rotations for physiotherapy students (PT), occupational therapy students (OT), and nurses studying at an advanced level to become primary health care nurses (PHCNs).

For peer review only - http://bmjopen.bmj.com/site/about/guidelines.xhtml

For peer review only

The learning environment is characterized by care activities of acute and non-acute visits, short and long patient encounters in the PHC, care during transports between different healthcare facilities, and patients seeking care for all varieties of complaints and acuity in the EMS. Registered nurses are clinical supervisors and teachers for both medical and nursing students in EMS and PHC. In the context of this study setting, the collaboration between the PHC and the EMS primarily revolves around the handover of patients suffering from acute illness and the need to transport the patient from the PHC to acute care.

Developing the interprofessional learning activity and the scenario used

During clinical rotations in the EMS and PHC, the students are situated in different geographical locations, therefore the clinical teachers and the research group in this study determined that the IPL activity should be carried out in a digital form. In addition, using a digital format for the IPL activity increased the possibilities for students to participate and increased environmental sustainability by reducing unnecessary travel. The scenario was developed collaboratively by clinical teachers and researchers. The content of the scenario included a situation where different healthcare professionals cared for the patient the handover of the patient in an acute situation, and patient safety in the context of PHC and the EMS. The scenario was built on (a) evidence-based guidelines [22, 23], (b) perspectives of person-centred care [24], (c) the Situation-Background-Assessment-Recommendation (SBAR) communication tool [25], and (d) crew resource management (CRM) [26]. Short sequences of the film were recorded with a smartphone used to illustrate different aspects of the care and handover in the scenario. The IPL activity was completed within 150 minutes, including a 15-minute break.

For peer review only - http://bmjopen.bmj.com/site/about/guidelines.xhtml

Data collection and participants

The digital IPL activities were scheduled when there were eligible students from different educational programs at their clinical rotations in the PHC or the EMS. The students were invited to the IPL activity thru by e-mail and/or electronic bulletin boards and had no previous relationship with the researchers (VL, MT, HC) but with some of the facilitators as they also worked as clinical teachers (NÅ, SG). Data collection took place on four different occasions, one time during the spring of 2021 and three times during the fall of 2021. A combination of data sources was used to capture the participant's experiences in and on the activity. The students and facilitators' reflective interplay during the learning activity and group discussions after the IPL activity were collected (total of 12 hrs of recording) using the digital platform recording function (image and sound). After each IPL activity, separate group discussions were held with the facilitation team. Data collection from the student and facilitator group discussions began with an open-ended question ("What was your experience of this IPL activity?" versus "What did you think about this activity?"). The facilitator's reflective notes were collected after each activity.

In total 22 students were consecutively included in the study, and seven facilitators and two observers participated in the IPL activity. In the spring of 2021, the scenario was first piloted with eight students, three interprofessional facilitators and two observers. The observers in this first scenario took notes on body language, group dynamics, and reflections. The results of the analysis of the pilot led to minor adjustments with the addition of a normal ECG and reminders in the PowerPoint to discuss CMR and SBAR. After piloting, three additional activities with 14 students from five educational programs (medical, nursing, PT, OT, and PHCN) were performed during the fall of 2021. In each activity, three facilitators participated. The interprofessional team of facilitators consisted of clinical teachers with previous

For peer review only - http://bmjopen.bmj.com/site/about/guidelines.xhtml

experiences in IPL activities: two physical therapists, one specialist nurse with extensive expertise in EMS, one specializing in paediatrics, one in primary healthcare, and one physician. Participating facilitators in the activities were determined by the opportunity to participate. The recordings were transcribed verbatim, and the reflective notes were electronically transcribed.

Analysis

The qualitative were analysed thematically [36]. The analysis was conducted following the steps described by Kiger and Varpio [27]. Initially, the transcribed text and field notes from the four IPL activities were read multiple times to gain familiarity with the collected material. After this, initial codes were identified from the texts, and followed by a search for preliminary themes by sorting the identified codes. The preliminary themes were then refined and labeled. Finally, the analysis concluded with the writing of the results. Each step was initiated by the last author and then reviewed by the co-authors. Any differences in interpretation were resolved through discussion until a consensus was reached. The process was iterative; a continuous movement between the codes, preliminary themes, and themes was made to preserve the essence of the collected data.

Results

Three themes describing the feasibility of the IPL activity from both the students' and facilitators' perspectives were constructed through the analysis: [1]*Interprofessional learning through collaborative reasoning in an authentic scenario,* [2] *Sharing a common model by communicating information in a structured way,* and [3] *reaching an understanding through collaborative reasoning*.

For peer review only - http://bmjopen.bmj.com/site/about/guidelines.xhtml

Interprofessional learning through collaborative reasoning in an authentic scenario

All participants evaluated the used scenario as authentic and based on the patients' needs during handover in an acute situation and patient safety. The students perceived the activity as a beneficial way of analysing the patient's changing needs between and with different professions throughout the whole chain of care as they normally did not know what happened with the patient before and after a care encounter. In addition, the students discussed that the developed scenario helped the students to discuss follow-up care at PHC after an acute illness and the support needed for the patient's family.

"...a strength of today's seminar was that many different aspects of care including discussions on the follow-up of the patient became clear..." (nurse student, activity #3).

Students agreed that all healthcare professionals need to know the handling of acute situations for example the scenario of cardiopulmonary resuscitation. To enhance IPL during clinical rotations, students and facilitators also discussed possibilities of developing scenarios by incorporating new film sequences into the scenario, for example by changing the outcome for the patient and thereby having the possibility to include other students in the IPL activity.

"*Think about it, adding another outcome for the patient in the scenario can make the scenario relevant for other students e.g., dietician or maybe homecare personnel* (nurse facilitator 1, activity #3).

The facilitators, students, and observers emphasized that sometimes the medical focus took over the discussions between the students. Facilitators explained this for several reasons. The first reason for that could be the fact that not all care aspects were explicitly presented in the

For peer review only - http://bmjopen.bmj.com/site/about/guidelines.xhtml

scenario and therefore were not discussed or analysed, and the second reason was associated with the facilitators themselves.

"….*XXXX* [name of facilitator] *needs to take a step back, keep quiet and wait for the students to talk, discuss and analyse all different aspects of the care…*" (nurse facilitator 3, activity #2).

An additional reason contributing to the dominance of medical focus was the composition of the group: when the group consisted of more medical students than nursing students the discussions had more of a medical perspective, while when there were more OT or PT students in the group their perspective of care was discussed to a greater extent and the medical issues in the scenario needed to be clarified more. During the scenario, the facilitators created a space for the students to take control, verbalize, and analyse together by listening, confirming, and adding critical questions when needed. The facilitators encouraged the students to explain and describe their professional aspects when caring for the patient. Facilitators experienced initial uncertainty among the students and guided them to communicate by open and de-dramatizing questions such as: *Do you have any experience with something similar*? … *What good reflection do you have, anyone else? Don't think about right or wrong answers; start reasoning, and together we will find the solution"* (PT facilitator 2 & nurse facilitator 3, activity #2,3,4)

Sharing a common model by communicating information in a structured way

The students discussed how the scenario highlighted the importance of communication in an acute situation and sharing information in a structured way using SBAR. They described that

For peer review only - http://bmjopen.bmj.com/site/about/guidelines.xhtml

structured way as a contributor to reaching a shared and patient-centred mental model for sharing patient information.

" *I know what to say and the other person knows what to expect*" (PT student, activity #2).

The shared information is depending, on the other hand, on who they are communicating with:

" *You need to be an active listener... it doesn't matter what I say if they don't listen*" (PHCN student, activity #4).

The common experience among facilitators, observers, and students was that using CMR for discussing leadership and responsibilities in care and handover situations supported the students' learning of who is leading and who is responsible. A drawn conclusion was that situated leadership and responsibility will be taken by the person with the best knowledge of handling an acute situation regardless of professionally situated leadership.

"The one with the most experience and knowledge needs to take the lead in a cardiac arrest situation… I may not have the best knowledge…" (medical student, activity #2) and a PT student continued in the same opinion: *"How to use everybody in a team when caring for a patient with a time-critical illness is essential*" (PT student, activity #1).

Reaching an understanding through collaborative reasoning

According to students, the developed IPL activity and scenario supported both wider understanding and new knowledge via interprofessional collaborative reasoning. They learned from each other about both the chain of care and other professions' roles and responsibilities while thinking and speaking from their perspective and listening to others in

For peer review only - http://bmjopen.bmj.com/site/about/guidelines.xhtml

planning care and medical treatment in both EMS and the PHC. One medical student expressed it as follows:

"The patient had been somewhere else before entering the PHC…", "It [the scenario] had a holistic perspective" …even though I know the physiotherapist profession I did not understand their roles and responsibilities in the chain of care" (medical student activity #2).

The students experienced the benefits of solving problems in an interprofessional team instead of doing it by themselves or with peers, as nurse students (activity #2) describe:

"…reasoning with different professions gave me more knowledge about what everyone can do and did in the care of the patient" (nurse student, activity #2).

IPL in digital form added, as students described, a new dimension and possibility to learn about each other's professions, knowledge, roles, and responsibilities in different contexts without needing to go between different healthcare facilities. By verbing their perspectives listening to others and sharing knowledge and experiences students concluded regarding patient care. It can be illustrated by the following quotation:

"To do it [Scenario] *interprofessional adds another dimension… that is very good… instead of just solving the case with my study colleagues" … "... everyone has shared their knowledge... I have listened...and reflected on how to act in a similar situation in my context"* (OT student, activity #3).

Nurse student discussed that both CRM and SBAR also supported interprofessional reasoning when discussing the care for the patient during the different transitions:

For peer review only - http://bmjopen.bmj.com/site/about/guidelines.xhtml

"I have little experience of the emergency care... the discussions have been good to prepare myself... knowledge about whom I should turn to, who has the knowledge, what information I should pass on, what will they want to know" (nurse student, activity #4).

The facilitators addressed the gap in the discussion when not all healthcare professionals were represented in the group of students who participated in the current study. In such cases, the facilitators took an active part in the discussion using their own clinical experiences. They contributed with their perspectives and knowledge to facilitate IPL and collaborative learning, including their learning from both the students and the other participating facilitators. However, the facilitators' participation was an act of balancing as the activity was developed for the students and they needed the space to discuss and solve the case in collaboration with the other students.

Discussion

Overall, the study findings show that the digital IPL activity and scenario were feasible and acceptable for enhancing IPL related to acute situations, handover, and patient safety. The findings show that the digital IPL activity may have a positive impact on students' knowledge of and understanding how to handle an acute care situation and of the value of collaborative reasoning in EMS and PHC settings. The scenario used in the IPL activity broadened the students´ perspective on the chain of care and enhanced discussions and knowledge of various professions' roles and responsibilities. The use of a digital format of an IPL activity allowed learning but also underscored the significance of interprofessional problem-solving within an acute care situation for the students. The findings also showed that when the activity was considered authentic, this enabled the students to communicate and share information and enabled an interprofessional analysis. Therefore, it is reasonable to

For peer review only - http://bmjopen.bmj.com/site/about/guidelines.xhtml

assume that the developed IPL activity supported students’ learning through an interactive group process consisting of collaborative analysis and joint critical reflections, factors that are also described by Reeves et.al. [2] to support IPL. However, there is a need to balance the different professions in their interactions and for the students to maintain their communication while facilitating learning and supporting interprofessional discussions [1, 4, 8]. With all the different healthcare professionals represented in the current IPL activity, the scenario covered different perspectives to achieve an interprofessional perspective on patient care through the entire care process. However, the different group compositions that could not be predicted before each IPL activity clarified the need for a scenario that could be used regardless of which professions participated in the IPL activity. Overall, it seemed that the digital IPL activity with a scenario, including a patient with an acute illness, was feasible and relevant for all healthcare professionals as they should be able to initiate care in an acute situation. However, out of this study, we do not know the effects of the uneven distribution among different health professions in the IPL activity; facilitators became more active and participated in the discussions by using their clinical profession when necessary. According to both facilitators and students, the facilitators had a direct impact on how the IPL activity was carried out. Students expressed a desire for more guidance and support from the facilitators, while facilitators recognized the importance of incorporating various professions' knowledge and perspectives into the clinical reasoning process for discussing optimal patient care. During the IPL activity, the facilitators employed different strategies to enhance the IPL. These strategies included encouraging active student participation, contributing additional knowledge to the scenario and discussions, asking questions to promote reflection, and guiding students in summarizing the discussions and patient findings using e.g., SBAR. Used facilitation strategies aligned with strategies identified in the Evans et.al. review [28]. The

For peer review only - http://bmjopen.bmj.com/site/about/guidelines.xhtml

facilitators in the study did not represent the various disciplines of students participating in all IPL activities, as suggested in previous literature [29]. When the facilitators did not represent all disciplines of students in the activity, they met before, after, during breaks, and sent text messages during each activity to ensure that perspectives from all healthcare professionals were considered and discussed during the scenario. In addition, the meetings aimed to adjust the facilitators' different roles and strategies as suggested in a previous study [30]. It may be considered important that the facilitator's healthcare professions represent all the various disciplines of students participating in an IPL activity, but it may also be of more importance that the students themselves represent the different disciplines in the discussions. However, further research is needed to clarify whether facilitators need to represent all the various disciplines of students participating in an IPL activity.

Conclusion

The main findings of this study showed that the digital IPL activity and scenario developed were feasible for enhancing IPL related to acute situations, handover, and patient safety. The positive impact of IPL concerning understanding and knowledge of different professions' perspectives on care and treatment in acute situations, particularly in the EMS and PHC context was evident. The findings also underscored the central role of facilitators in promoting collaborative reasoning, emphasizing the need for a balanced and inclusive approach for optimal IPL outcomes. By using digital solutions for the IPL activity, the EMS can offer IPL activities in collaboration with another healthcare setting for students doing their clinical rotations in the EMS and PHC.

For peer review only - http://bmjopen.bmj.com/site/about/guidelines.xhtml

Limitations

The study has several limitations. Firstly, the number of participants and completed IPL activities could be considered as low. All students doing their clinical rotation in the PHC or the EMS when the activities were conducted were invited by e-mail and/or electronic bulletin board , but we do not know if all eligible students were reached. Secondly, not all professions among the students were representative during IPL activities. This was adjusted for by the facilitators taking an active professional role in the IPL activity, which may have caused a decrease in students' participation in the discussions. However, this was not obvious during the analysis, but we do not know how the facilitators affected the IPL when the students did not represent all healthcare professionals. The third limitation of this study may have been the authors' preconceptions and personal experiences may have influenced the interpretation and understanding of the study. The fact that the authors are female and have extensive clinical experience in the EMS or the PHC, and the fifth author is an intensive care nurse and IPL promotor, may introduce biases during the study design, data collection, and analysis. Despite the potential for bias, the authors' preconceptions can also be seen as a strength as they contribute to interpreting and understanding the study findings. Despite these limitations, this is the first study using data from IPL learning activities with students in clinical rotations in both the PHC and the EMS. Another strength of this study is that for the students it was reasonable to assume that the IPL activity gave the students a possibility for IPL from the perspective of patient safety and patient needs in an acute situation. The third strength of the current study are multiple data sources (interviews, notes, observations) and 31 participants creating new knowledge based on qualitative analysis. However quantitative and qualitative evaluation is warranted of IPL learning activities with authentic scenarios in acute

For peer review only - http://bmjopen.bmj.com/site/about/guidelines.xhtml

transition situations over a longer period of clinical rotations and with a higher number of students.

Abbreviations

AMI	Acute Myocardial Infarction
CRM	Crew resource management
ECG	Electrocardiography
IPL	Interprofessional learning
OT	Occupational therapy
PHC	Primary health center
PHCN	Primary health care nurse
PT	Physiotherapy
SBAR	Situation-Background-Assessment-Recommendation

Declarations

Ethics approval and consent to participate

This study was approved by Etikprövnings myndigheten (The ethics review authority), Sweden (No:2021-00586). Ethical principles in accordance with the Declaration of Helsinki were followed [31]. Participants received both oral and written information about the study describing the aim of the study and stating that their participation was voluntary and that they could withdraw at any time without explanation. Informed consent was obtained from all participants in the study.

Consent for publication

Not applicable

Availability of data and materials

For peer review only - http://bmjopen.bmj.com/site/about/guidelines.xhtml

The data analyzed during the current study are not publicly available due to the participants individual privacy could be compromised but are available in Swedish from the corresponding author on reasonable request.

Competing interests

The authors declare no conflicts of interest

Funding

Karolinska Institutet, Stockholm Sweden (educational funds for employees no: 20200792) and Region Stockholm (ALF project no: 20200223). No person from the founding committee participated in design of the study, data collection, analysis of data or in writing the manuscript.

Author contributions

The first, second and last authors contributed during the study planning. VL, NÅ, SG collected data and VL initiated data analysis, HC and MT supported the data analysis. MT organized and coordinated learning activities at the Academic primary health care center. VL drafted the manuscript and all authors contributed, read, and approved the final manuscript.

Acknowledgements

Thank you to all the students who participated in this study, Kristina Björk sharing knowledge on care in the ambulance service, academic clinical adjuncts Helena Solman, Hege Jahr and Abdullah Almaasarani.

Authors' information

For peer review only

VL, professor at Umeå University and senior lecture at Karolinska Institutet. VL also works as a RN and supervising students in the ambulance service, Samariten Ambulance. HC, intensive care nurse, senior lecture, and IPL promotor at Karolinska Institutet. NÅ physiotherapist at Jakobsbergs rehabilitation clinic and SG, specialist nurse in pediatrics at Vällingby Child Health Care Center, both working in primary health care and are academic clinical adjunct and facilitators from Academic primary health care center and MT are associate professor at KI and an academic coordinator of on-site training where theoretical knowledge in pedagogy integrates with professional skills within primary healthcare practice for students, research, and continuing education for healthcare providers.

References

1. Conte H, Wihlborg J, Lindström V: **Developing new possibilities for interprofessional learning- students' experience of learning together in the ambulance service**. *BMC Med Educ* 2022, **22**(1):192.
2. Reeves S, van Soeren M, Macmillan K, Zwarenstein M: **Medicine and nursing: a social contract to improve collaboration and patient-centred care?** *J Interprof Care* 2013, **27**(6):441-442.
3. CAIPE.: **Statement of Purpose CAIPE 2016**. 2016.
4. Conte H, Jirwe M, Scheja M, Hjelmqvist H: **Get it together: Issues that facilitate collaboration in teams of learners in intensive care**. *Med Teach* 2016, **38**(5):491-497.
5. Reeves S, Fletcher S, Barr H, Birch I, Boet S, Davies N, McFadyen A, Rivera J, Kitto S: **A BEME systematic review of the effects of interprofessional education: BEME Guide No. 39**. *Med Teach* 2016, **38**(7):656-668.
6. O'Leary N, Salmon N, Clifford A, O'Donoghue M, Reeves S: **'Bumping along': a qualitative metasynthesis of challenges to interprofessional placements**. 2019, **53**(9):903-915.
7. Oosterom N, Floren LC, Ten Cate O, Westerveld HE: **A review of interprofessional training wards: Enhancing student learning and patient outcomes**. *Med Teach* 2019, **41**(5):547-554.
8. Croker A, Fisher K, Smith T: **When students from different professions are co-located: the importance of interprofessional rapport for learning to work together**. 2015, **29**(1):41-48.
9. Visser CLF, Ket JCF, Croiset G, Kusurkar RA: **Perceptions of residents, medical and nursing students about Interprofessional education: a systematic review of the quantitative and qualitative literature**. *BMC Med Educ* 2017, **17**(1):77.
10. **Patient Safety** [https://www.who.int/news-room/fact-sheets/detail/patient-safety]
11. McCoy L, Lewis JH, Simon H, Sackett D, Dajani T, Morgan C, Hunt A: **Learning to Speak Up for Patient Safety: Interprofessional Scenarios for Training Future Healthcare Professionals**. *J Med Educ Curric Dev* 2020, **7**:2382120520935469.
12. Young J, Zolio L, Brock T, Harrison J, Hodgkinson M, Kumar A, Morphet J, Kent F: **Interprofessional learning about medication safety**. *Clin Teach* 2021, **18**(6):656-661.

For peer review only - http://bmjopen.bmj.com/site/about/guidelines.xhtml

13. Wood K, Crouch R, Rowland E, Pope C: **Clinical handovers between prehospital and hospital staff: literature review**. *Emerg Med J* 2015, **32**(7):577-581.
14. **Sentinel Event Data Summary- General Information & 2021 Update** [https://www.jointcommission.org/-/media/tjc/documents/resources/patient-safety-topics/sentinel-event/sentinel-event-general-information-and-2021-update.pdf]
15. Campbell B, Stirling C, Cummings E: **Continuity matters: Examining the 'information gap' in transfer from Residential Aged Care, ambulance to emergency triage in southern Tasmania**. *Int Emerg Nurs* 2017, **32**:9-14.
16. Cantero-López N, González-Chordá VM, Valero-Chillerón MJ, Mena-Tudela D, Andreu-Pejó L, Vila-Candel R, Cervera-Gasch Á: **Attitudes of Undergraduate Nursing Students towards Patient Safety: A Quasi-Experimental Study**. *Int J Environ Res Public Health* 2021, **18**(4).
17. Leape L, Berwick D, Clancy C, Conway J, Gluck P, Guest J, Lawrence D, Morath J, O'Leary D, O'Neill P *et al*: **Transforming healthcare: a safety imperative**. *Qual Saf Health Care* 2009, **18**(6):424-428.
18. Mulholland P, Barnett T, Spencer J: **Interprofessional learning and rural paramedic care**. *Rural Remote Health* 2014, **14**(3):2821.
19. Mulholland P, O'Meara P, Walker J, Stirling C, Tourle V: **Multidisciplinary Practice in Action: The Rural Paramedic – It's Not Only Lights and Sirens**. *Australasian Journal of Paramedicine* 2009, **7**(2).
20. Bowen DJ, Kreuter M, Spring B, Cofta-Woerpel L, Linnan L, Weiner D, Bakken S, Kaplan CP, Squiers L, Fabrizio C *et al*: **How we design feasibility studies**. *Am J Prev Med* 2009, **36**(5):452-457.
21. Tong A, Sainsbury P, Craig J: **Consolidated criteria for reporting qualitative research (COREQ): a 32-item checklist for interviews and focus groups**. *Int J Qual Health Care* 2007, **19**(6):349-357.
22. Levine GN, Bates ER, Blankenship JC, Bailey SR, Bittl JA, Cercek B, Chambers CE, Ellis SG, Guyton RA, Hollenberg SM *et al*: **2015 ACC/AHA/SCAI Focused Update on Primary Percutaneous Coronary Intervention for Patients With ST-Elevation Myocardial Infarction: An Update of the 2011 ACCF/AHA/SCAI Guideline for Percutaneous Coronary Intervention and the 2013 ACCF/AHA Guideline for the Management of ST-Elevation Myocardial Infarction**. *J Am Coll Cardiol* 2016, **67**(10):1235-1250.
23. Soar J, Böttiger BW, Carli P, Couper K, Deakin CD, Djärv T, Lott C, Olasveengen T, Paal P, Pellis T *et al*: **European Resuscitation Council Guidelines 2021: Adult advanced life support**. *Resuscitation* 2021, **161**:115-151.
24. **About person-centred care** [https://www.gu.se/en/gpcc/about-person-centred-care]
25. Randmaa M, Mårtensson G, Leo Swenne C, Engström M: **SBAR improves communication and safety climate and decreases incident reports due to communication errors in an anaesthetic clinic: a prospective intervention study**. *BMJ Open* 2014, **4**(1):e004268.
26. Weller J, Boyd M, Cumin D: **Teams, tribes and patient safety: overcoming barriers to effective teamwork in healthcare**. *Postgrad Med J* 2014, **90**(1061):149-154.
27. Kiger ME, Varpio L: **Thematic analysis of qualitative data: AMEE Guide No. 131**. *Medical Teacher* 2020, **42**(8):846-854.
28. Evans SM, Ward C, Reeves S: **Online interprofessional education facilitation: A scoping review**. *Medical Teacher* 2019, **41**(2):215-222.
29. Burgess A, Kalman E, Haq I, Leaver A, Roberts C, Bleasel J: **Interprofessional team-based learning (TBL): how do students engage?** 2020, **20**(1):118.
30. Ruiz MG, Ezer H, Purden M: **Exploring the nature of facilitating interprofessional learning: findings from an exploratory study**. *J Interprof Care* 2013, **27**(6):489-495.
31. **World Medical Association-WMA Declaration of Helsinki – Ethical principles for medical research involving human subjects**

For peer review only

COREQ (COnsolidated criteria for REporting Qualitative research) Checklist

A checklist of items that should be included in reports of qualitative research. You must report the page number in your manuscript where you consider each of the items listed in this checklist. If you have not included this information, either revise your manuscript accordingly before submitting or note N/A.

Topic	Item No.	Guide Questions/Description	Reported on Page No.
**Domain 1: Research team and reflexivity**			
Personal characteristics			
Interviewer/facilitator	1	Which author/s conducted the interview or focus group?	
Credentials	2	What were the researcher's credentials? E.g. PhD, MD	
Occupation	3	What was their occupation at the time of the study?	
Gender	4	Was the researcher male or female?	
Experience and training	5	What experience or training did the researcher have?	
Relationship with participants			
Relationship established	6	Was a relationship established prior to study commencement?	
Participant knowledge of the interviewer	7	What did the participants know about the researcher? e.g. personal goals, reasons for doing the research	
Interviewer characteristics	8	What characteristics were reported about the inter viewer/facilitator? e.g. Bias, assumptions, reasons and interests in the research topic	
**Domain 2: Study design**			
Theoretical framework			
Methodological orientation and Theory	9	What methodological orientation was stated to underpin the study? e.g. grounded theory, discourse analysis, ethnography, phenomenology, content analysis	
Participant selection			
Sampling	10	How were participants selected? e.g. purposive, convenience, consecutive, snowball	
Method of approach	11	How were participants approached? e.g. face-to-face, telephone, mail, email	
Sample size	12	How many participants were in the study?	
Non-participation	13	How many people refused to participate or dropped out? Reasons?	
Setting			
Setting of data collection	14	Where was the data collected? e.g. home, clinic, workplace	
Presence of non-participants	15	Was anyone else present besides the participants and researchers?	
Description of sample	16	What are the important characteristics of the sample? e.g. demographic data, date	
Data collection			
Interview guide	17	Were questions, prompts, guides provided by the authors? Was it pilot tested?	
Repeat interviews	18	Were repeat inter views carried out? If yes, how many?	
Audio/visual recording	19	Did the research use audio or visual recording to collect the data?	
Field notes	20	Were field notes made during and/or after the inter view or focus group?	
Duration	21	What was the duration of the inter views or focus group?	
Data saturation	22	Was data saturation discussed?	
Transcripts returned	23	Were transcripts returned to participants for comment and/or	

For peer review only

Topic	Item No.	Guide Questions/Description	Reported on Page No.
		correction?	
**Domain 3: analysis and findings**			
Data analysis			
Number of data coders	24	How many data coders coded the data?	
Description of the coding tree	25	Did authors provide a description of the coding tree?	
Derivation of themes	26	Were themes identified in advance or derived from the data?	
Software	27	What software, if applicable, was used to manage the data?	
Participant checking	28	Did participants provide feedback on the findings?	
Reporting			
Quotations presented	29	Were participant quotations presented to illustrate the themes/findings? Was each quotation identified? e.g. participant number	
Data and findings consistent	30	Was there consistency between the data presented and the findings?	
Clarity of major themes	31	Were major themes clearly presented in the findings?	
Clarity of minor themes	32	Is there a description of diverse cases or discussion of minor themes?	

Developed from: Tong A, Sainsbury P, Craig J. Consolidated criteria for reporting qualitative research (COREQ): a 32-item checklist for interviews and focus groups. *International Journal for Quality in Health Care*. 2007. Volume 19, Number 6: pp. 349 – 357

Once you have completed this checklist, please save a copy and upload it as part of your submission. DO NOT include this checklist as part of the main manuscript document. It must be uploaded as a separate file.

For peer review only

Facilitating Interprofessional learning: Experiences of using a digital activity for training handover of critically ill patients between a Primary Health Care Centre and Ambulance Services – a qualitative study

Journal:	*BMJ Open*
Manuscript ID	bmjopen-2023-083585.R1
Article Type:	Original research
Date Submitted by the Author:	15-Apr-2024
Complete List of Authors:	Taloyan, Marina; Karolinska Institute, Department of Neurobiology, Care Sciences and Society, Division of Family Medicine and Primary Care; Region Stockholm, Academic primary health care center<br>Helen, Conte; Karolinska Institutet, Department of Neurobiology, Care Sciences and Society, Division of Nursing<br>Ninni, Åkesson; Region Stockholm , Rehab North West<br>Guldbrand, Sofie; Region Stockholm, Child Health Care Centre<br>Lindström, Veronica; Umeå University, Department of Nursing and the Ambulance Service, Västerbotten; Sophiahemmet Hogskola, Department of Health Promotion Science
**Primary Subject Heading**:	Medical education and training
Secondary Subject Heading:	Emergency medicine, Qualitative research, Nursing, Medical education and training
Keywords:	MEDICAL EDUCATION & TRAINING, ACCIDENT & EMERGENCY MEDICINE, Primary Health Care, Feasibility Studies, QUALITATIVE RESEARCH

BMJ

I, the Submitting Author has the right to grant and does grant on behalf of all authors of the Work (as defined in the below author licence), an exclusive licence and/or a non-exclusive licence for contributions from authors who are: i) UK Crown employees; ii) where BMJ has agreed a CC-BY licence shall apply, and/or iii) in accordance with the terms applicable for US Federal Government officers or employees acting as part of their official duties; on a worldwide, perpetual, irrevocable, royalty-free basis to BMJ Publishing Group Ltd ("BMJ") its licensees and where the relevant Journal is co-owned by BMJ to the co-owners of the Journal, to publish the Work in this journal and any other BMJ products and to exploit all rights, as set out in our licence.

The Submitting Author accepts and understands that any supply made under these terms is made by BMJ to the Submitting Author unless you are acting as an employee on behalf of your employer or a postgraduate student of an affiliated institution which is paying any applicable article publishing charge ("APC") for Open Access articles. Where the Submitting Author wishes to make the Work available on an Open Access basis (and intends to pay the relevant APC), the terms of reuse of such Open Access shall be governed by a Creative Commons licence – details of these licences and which Creative Commons licence will apply to this Work are set out in our licence referred to above.

Other than as permitted in any relevant BMJ Author's Self Archiving Policies, I confirm this Work has not been accepted for publication elsewhere, is not being considered for publication elsewhere and does not duplicate material already published. I confirm all authors consent to publication of this Work and authorise the granting of this licence.

For peer review only

Begränsad delning

Facilitating Interprofessional learning: Experiences of using a digital activity for training handover of critically ill patients between a Primary Health Care Centre and Ambulance Services – a qualitative study

Authors: Taloyan Marina[1,2*] Conte Helen[3*,] Åkesson Ninni[4], Guldbrand Sofie[5], Lindström Veronica[6,7]

Affiliations:

[1]Academic primary health care centre, Region Stockholm, Sweden

[2]Department of Neurobiology, Care Sciences and Society, Division of Family Medicine and Primary Care, Karolinska Institutet, Stockholm, Sweden

[3]Department of Neurobiology, Care Sciences and Society, Section of Nursing, Karolinska Institutet, Stockholm, Sweden

[4] Rehab North West, Region Stockholm, Sweden

[5] Child Health Care Centre, Region Stockholm; Sweden

[6]Department of Nursing, Umeå University & Department of Ambulance Service, Region Västerbotten, Umeå, Sweden.

[7] Department for Health Promotion Science, Sophiahemmet University, Stockholm, Sweden.

[*] co-first authorship

Word count: 6663

Corresponding author:
Veronica Lindström
Orcid ID: 0000-0003-1386-3203

Department of Nursing, Umeå University & Department of Ambulance Service, Region Västerbotten, Umeå, Sweden.
e-mail: veronica.lindstrom@umu.se
Telephone: +4670-3428157

For peer review only

Begränsad delning

Abstract

Objective: To explore students' and facilitators' experiences of using a developed digital activity for interprofessional learning (IPL) focusing on critically ill patient handovers from a Primary Health Care (PHC) centre to the ambulance service. **Design**: A qualitative study design was employed, and the reporting of this study adheres to the COREQ guidelines for qualitative studies. **Setting:** A PHC centre and the ambulance service in Stockholm, Sweden **Participants:** A total of 31 participants were included in the study, 22 students from five different healthcare professions, seven facilitators, and two observers. **Intervention:** A digital IPL activity was developed to overcome geographical distances and the scenario included the handover of a critically ill patient from personnel within the PHC centre to the ambulance service personnel for transport to an emergency department. Four digital IPL activities were conducted in 2021. **Results:** The digital IPL activity eliminated the issue of geographical distance for students and facilitators, and it enabled the students to find an interprofessional model for collaboration through reasoning, by communicating and sharing knowledge with the support of a common structure. Participants perceived the digital IPL activity and scenario as authentic, feasible, and facilitated IPL. Using a case with an acute and life-threatening condition was a success factor for students to experience high realism in their IPL on patient safety, handover, care, and treatment**. Conclusion:** The developed digital IPL activity facilitated the students' IPL and demonstrated potential sustainability as the digital approach supported overcoming geographical distances for both students and facilitators. By using a scenario involving an authentic case focusing on handovers of a critically ill patient, IPL, feasibility, and acceptability were supported. However, a comprehensive evaluation, both quantitative and qualitative, over an extended period of clinical rotations and involving a larger group of students is warranted.

For peer review only

Keywords: Feasibility**,** Interprofessional learning, Interprofessional education, Patient safety; Digital learning activity; Chain of care; Emergency Medical Service; Primary healthcare care service

Strengths and limitations of this study (five bullet points)

- To our knowledge, this study is the first to collect data from digital IPL learning activities with students in clinical rotations in both the Primary Health Care Centre and the ambulance service.
- Multiple data sources (interviews, notes, observations) and 31 participants creating new knowledge based on qualitative analysis.
- The number of participants and completed IPL activities could be considered low and we do not know if all eligible students were reached.
- Not all professions were representative among the students during all IPL activities.
- The authors' preconceptions and personal experiences may have influenced the interpretation and understanding of the study.

Introduction

It is essential to establish interprofessional learning (IPL) activities that ensure high-quality education. This is particularly critical in clinical settings where IPL is still in the early stages of development. In the ambulance service, IPL is not yet firmly established. Historically, the ambulance service has been perceived as suboptimal for undergraduate students' clinical rotations, primarily due to the services' previous function of responding to emergency calls and conveying sick or injured individuals to hospitals. However, as the ambulance service has

For peer review only

Begränsad delning

developed into a recognized discipline of pre-hospital emergency care delivering advanced medical interventions and care [1], it has been shown that within the ambulance service, students can gain proficiency in nursing, medical, and collaborative skills [2]. As the number of undergraduate students completing clinical rotations in the ambulance service has increased, so have the expectations for activities to be conducted to support the students' IPL. Therefore, a need to develop and systematically evaluate easily accessible IPL activities suitable for students doing their clinical rotations in the ambulance service was identified. Different IPL activities have been developed and evaluated over the years but the setting and context matter [3] and, contextual factors influence IPL [4]. Therefore, this study aimed to explore students' and facilitators' experiences of using a developed digital activity for interprofessional learning (IPL) focusing on critically ill patient handovers from a Primary Health Care (PHC) centre to the ambulance service.

Background

IPL includes a range of activities depending on goals and desired outcomes, e.g., short digital seminars or shadowing where the students' learning originates from interactive group processes including collaborative analysis and joint critical reflection [5]. The students are active participants and learn with and from each other in authentic and patient-centred activities [6]. They negotiate their different experiences to fully understand their role and the roles of other professions [7]. However, finding places, spaces, and time for students from different educational programs to learn together in healthcare can be a challenge since education is not always situated in contexts that support IPL [8]. Environments and care activities that naturally could support IPL are situated during clinical rotations where students from different educational programs collaborate in patient care [9, 10]. Students acknowledged that experiencing a diversity of IPL activities during clinical rotations is vital for

For peer review only

For peer review only - http://bmjopen.bmj.com/site/about/guidelines.xhtml

learning how to work together [11]. Co-location is important but not enough for different professions to learn together [11]. Facilitated learning is part of building interprofessional rapport in groups and balancing the participation of the different professions in their interactions is vital for learning together [2, 7, 11]. Students suggest that bringing a sense of their profession into the interactions and being inclusive of other professions are two key qualities for IPL [11] and that gaps in role conceptions are a crucial barrier [12] and could cause risks for patients.

The World Health Organization (WHO) states that healthcare services around the world should be safe, and person-centred [13]. Research has shown that different IPL activities can introduce patient safety concepts and experiential practice through interprofessional communication and analysis among students [14, 15]. To improve patient safety in the healthcare setting, training must start with students [16, 17]. Working as a professional in the ambulance service involves collaborating with various healthcare professionals when providing care for patients transported to emergency departments or when responding to incidents in home healthcare settings [18]. There is limited knowledge about how undergraduate students perceive digital IPL activities focusing on handovers of critically ill patients within the PHC centre. There is also limited knowledge about handovers of critically ill patients from the PHC centre to the ambulance service. The handover between different healthcare settings are complex process that includes communicating information between professionals responsible for the patient's care at different units or sites [19] and communication failures are one of the main causes of adverse events, particularly in handover situations [20]. When the handover is compromised, essential information can be altered or lost, transfer of cognitive bias may occur, patients may be exposed to adverse events and patient safety issues may occur [21]. Expanding IPL activities beyond organizational

For peer review only

Begränsad delning

boundaries to include the handover of patients between different healthcare organizations could offer a possibility for students to practice and learn handover and patient safety in a new context. The ambulance service collaborates with PHC and community-based care and is involved in both receiving and handing over patients within these organizations [22, 23]. To our knowledge, the handover of critically ill patients from the PHC centre to the ambulance service has not previously been utilized for IPL.

Methods

Design

A qualitative study design was used to collect and analyse both the students' and facilitators' experiences through their reflections on the newly developed digital IPL activity based on an authentic scenario. By examining the experiences of supervisors and students, conclusions can be reached regarding IPL in a sparsely explored IPL context, as well as the acceptability and feasibility of the developed digital IPL activity. Acceptability captures how the intended recipients react to the activity, i.e. is it appropriate, relevant, or sustainable [24], and feasibility captures if the developed digital IPL activity is sustainable for continual use in the setting. The consolidated criteria for reporting qualitative research COREQ guidelines were used to guide the study [25]. The study was approved by the ethics review authority in Sweden (No:2021-00586).

Patient and Public Involvement

No patient and public involvement

Setting

The ambulance service and the PHC centre provide clinical rotations for medical and nursing students. The PHC centre also provides clinical rotations for physiotherapy students (PT), occupational therapy students (OT), and nurses studying at an advanced level to become

For peer review only - http://bmjopen.bmj.com/site/about/guidelines.xhtml

primary health care nurses (PHCNs). In the PHC centre, the learning environment is characterized by care activities of acute and non-acute visits, short and long patient encounters and the students care for patients with all varieties of complaints. The learning environment varies between care for patients at their home and the clinic, some patients are followed up over time, and some only visit the clinic once. The ambulance service responds to emergency calls, and the students care for patients with all varieties of complaints and acuity. The learning environment in the ambulance service is characterized by short patient care encounters, care at patients' homes, at the scene of an injury, and during transports between different healthcare facilities. The team in the ambulance consists of an emergency medical technician with basic life support competence and a registered nurse (RN) with one year of additional training in emergency care [26]. The clinical supervisor for both medical and nursing students in the ambulance service is the RN. Collaboration between PHC centres and the ambulance service primarily revolves around the handover of patients suffering from acute illness and needing to be transported from the PHC centre to an emergency department. With two different healthcare organizations collaborating and a lack of co-location, a prerequisite for conducting the IPL activity was that they were conducted digitally. In addition, using a digital format for the IPL activity increased the possibilities for students to participate and increased environmental sustainability by reducing unnecessary travel.

Developing the interprofessional learning activity and the scenario used

The scenario was developed by facilitators and researchers who were clinicians working in the ambulance service, PHC centre, or intensive care unit. The content of the scenario included (1) situations where different healthcare professionals cared for the patient, (2) handovers of patients in acute situations, and (3) patient safety in the context of PHC and the ambulance service. The scenario was built on several sources: (1)evidence-based guidelines [27-29], (2)

For peer review only - http://bmjopen.bmj.com/site/about/guidelines.xhtml

Begränsad delning

perspectives of person-centered care [30], (3) the Situation-Background-Assessment-Recommendation (SBAR) communication tool [31], and finally (4) Crew Resource Management (CRM) [32]. After developing the scenario, short sequences were recorded with a smartphone to illustrate different aspects of the care and handover situations. To reduce costs, the patient was acted by one of the facilitators and the recording was made by a clinical colleague from the ambulance service. In total, approximately six working hours were required for the development, filming, and editing of the scenario and for embedding the material into a PowerPoint presentation. The scenario included critical events in the patient's symptom development when suffering from an acute myocardial infarction leading to a cardiac arrest and after advanced cardio-pulmonary resuscitation (CPR) return to spontaneous circulation. The scenario also included handover situations, together with supplementary information (e.g., vital signs and ECG). There was a balance between information and questions related to each healthcare profession, to enable the students to learn from each profession during the scenario. Questions about CRM and SBAR were added in connection to each care transition in the scenario. The scenario included three episodes of handovers: 1) physiotherapist – primary health care nurse 2) primary health care nurse or physician or physiotherapist – dispatcher at the emergency medical communication centre, 3) physiotherapist or primary care nurse or physician – ambulance personnel. The IPL activity was completed within 150 minutes, including a 15-minute break.

Data collection and participants

The study and data collection were done in Stockholm, Sweden where students did their clinical rotation in the ambulance service or at a PHC centre. The students invited to participate and evaluate the developed IPL activity were from three different higher education institutions. The digital IPL activities were scheduled when there were eligible students from

For peer review only

For peer review only - http://bmjopen.bmj.com/site/about/guidelines.xhtml

different educational programs at their clinical rotations in the PHC centre or the ambulance service at the same time. The students were invited to participate in the IPL activity through e-mail and/or electronic bulletin boards and had no previous relationship with the researchers (VL, MT, HC). Some of the facilitators had previous knowledge about the students as they also worked as clinical teachers (NÅ, SG) but they were not responsible for the students grading. Data collection took place on four different occasions, once during the spring of 2021 and three times during the fall of 2021.

A combination of data sources was used to capture the participant's experiences in and on the activity. The students' and facilitators' reflective interplay during the IPL activity and group discussions after the activity were collected (a total of 12 hrs of recording) using the digital platform recording function (image and sound). After each IPL activity, separate group discussions were held with the facilitation team. Data collection from the student and facilitator group discussions was initiated with an open-ended question ("What was your experience of this IPL activity?" versus "What did you think about this activity?"). The facilitator's reflective notes were collected after each IPL activity.

In total 22 students were consecutively included in the study, and seven facilitators and two observers participated in the IPL activity. In the spring of 2021, the scenario was first piloted with eight students, three interprofessional facilitators, and two observers. The observers in this first scenario took notes on body language, group dynamics, and reflections. The results of the analysis of the pilot led to minor adjustments with the addition of a normal ECG and reminders in the PowerPoint to discuss CMR and SBAR, the pilot was included in the analysis. After piloting, three additional activities with 14 students from five educational programs (medical, nursing, PT, OT, and PHCN) were performed during the fall of 2021. In each activity,

For peer review only - http://bmjopen.bmj.com/site/about/guidelines.xhtml

Begränsad delning

three facilitators participated. The interprofessional team of facilitators consisted of clinical teachers with previous experiences in IPL activities: two physical therapists, one specialist nurse with extensive expertise in the ambulance service, one specializing in pediatrics, one in primary healthcare, and one physician. Participating facilitators in the activities were determined by the opportunity to participate. The recordings were transcribed verbatim, and the reflective notes were electronically transcribed.

Analysis

The qualitative materials were analysed thematically and the analysis was conducted according to the steps described by Kiger and Varpio [33]. Initially, the transcribed text and field notes from the four IPL activities were read multiple times to gain familiarity with the collected material. After this, codes were identified from the texts, and this step was followed by a search and construction of preliminary themes by sorting the identified codes. The preliminary themes were then refined and labeled. In the final step, the analysis was concluded with the writing of the results. The process was iterative; a continuous movement between the codes, preliminary themes, and themes was made to preserve the essence of the collected data. Each step in the process was initiated by the last author and then reviewed by the co-authors. Any differences in interpretation were resolved through discussion until a consensus was reached.

Results

Three themes describing participants' experiences of IPL, the acceptability, and the feasibility of the developed and used IPL activity, from both the students' and facilitators' perspectives, were constructed through the analysis: [1]*Interprofessional learning through*

For peer review only - http://bmjopen.bmj.com/site/about/guidelines.xhtml

collaborative reasoning in an authentic scenario, [2] *Sharing a common model by communicating information in a structured way,* and [3] *reaching an understanding through collaborative reasoning*.

Interprofessional learning through collaborative reasoning in an authentic scenario

All participants evaluated the used scenario as authentic and based on the patients' needs during handover in an acute situation and patient safety. The students perceived the activity as a feasible and beneficial way of analysing the patient's changing problems and need between and with different professions throughout the whole chain of care. They normally did not know what happened with the patient either before or after a care encounter. In addition, the students discussed that the developed scenario helped the students to discuss follow-up care at PHC after an acute illness and the support needed for the patient's family.

"…a strength of today's seminar was that many different aspects of care including discussions on the follow-up of the patient became clear..." (nurse student, activity #3).

Students agreed that all healthcare professionals need to know how to handle acute situations for example the CPR scenario. To enhance IPL during clinical rotations, students and facilitators also discussed the feasibility and possibilities of developing scenarios for future digital IPL activities by incorporating new film sequences into the scenario, for example by changing symptom presentation, and/or changing the outcome for the patient and thereby having the possibility to include other students in the IPL activity.

For peer review only

Begränsad delning

"*Think about it, adding another outcome for the patient in the scenario can make the scenario relevant for other students e.g., dietician or maybe homecare personnel* (nurse facilitator 1, activity #3).

The facilitators, students, and observers emphasized that sometimes the medical focus took over the discussions between the students and risked reducing the possibilities for IPL. Facilitators explained several reasons for this. The first reason could be the fact that not all healthcare professionals and care aspects were explicitly presented in the scenario, and therefore, were not discussed or analysed. The second reason was associated with the facilitators themselves.

"*….XXXX* [name of facilitator] *needs to take a step back, keep quiet and wait for the students to talk, discuss and analyse all different aspects of the care…*" (nurse facilitator 3, activity #2).

An additional reason contributing to the medical focus was the composition of the group of participants: when the group consisted of more medical students than nursing students the discussions had more of a medical perspective, while when there were more OT or PT students in the group their perspective of care was discussed to a greater extent and the medical issues in the scenario needed to be clarified more. In these situations, the facilitators needed to ensure that all aspects were discussed to ensure IPL and the acceptability of the developed scenario. During the scenario, the facilitators created a space for the students to take control, verbalize, and analyse together by listening, confirming, and adding critical questions when needed. The facilitators encouraged the students to explain and describe their professional aspects when caring for the patient. Facilitators experienced initial uncertainty among the students and guided them to communicate by open and de-dramatizing questions such as:

For peer review only - http://bmjopen.bmj.com/site/about/guidelines.xhtml

Do you have any experience with something similar? … *What good reflection do you have, anyone else? Don't think about right or wrong answers; start reasoning, and together we will find the solution"* (PT facilitator 2 & nurse facilitator 3, activity #2,3,4)

Sharing a common model by communicating information in a structured way

The students discussed how the scenario highlighted the importance of communication in an acute situation and sharing patient information in a structured way using SBAR. They described that the structure contributed to reaching a shared and patient-centered mental model.

" *I know what to say and the other person knows what to expect*" (PT student, activity #2).

The shared information is depending, on the other hand, on who they are communicating with:

" *You need to be an active listener... it doesn't matter what I say if they don't listen*" (PHCN student, activity #4).

The common experience among facilitators, observers, and students was that using CMR for discussing leadership and responsibilities in care and handover situations supported the students' learning of who is leading and who is responsible for which part of the care action. A conclusion was that situated leadership and responsibility should be taken by the person with the best knowledge of handling an acute situation regardless of professionally situated leadership.

For peer review only

Begränsad delning

"The one with the most experience and knowledge needs to take the lead in a cardiac arrest situation… I may not have the best knowledge…" (medical student, activity #2) and a PT student continued in the same opinion: *"How to use everybody in a team when caring for a patient with a time-critical illness is essential*" (PT student, activity #1).

Reaching an understanding through collaborative reasoning

According to students, the developed IPL activity and scenario facilitated both wider understanding and new knowledge via interprofessional collaborative reasoning. They learned from each other about both the chain of care and other professions' roles and responsibilities while thinking and speaking from their perspective and listening to others in planning care and medical treatment in both the ambulance service and at the PHC centre. One medical student expressed it as follows:

"The patient had been somewhere else before entering the PHC…", "It [the scenario] had a holistic perspective" …even though I know the physiotherapist profession I did not understand their roles and responsibilities in the chain of care" (medical student activity #2).

The students experienced the benefits of solving problems in an interprofessional team instead of doing it by themselves or with peers, as nurse students (activity #2) describe:

"…reasoning with different professions gave me more knowledge about what everyone can do and did in the care of the patient" (nurse student, activity #2).

IPL in digital form added, as students described, a new dimension and facilitated learning about each other's professions, knowledge, roles, and responsibilities in different contexts without needing to go between different healthcare facilities. By verbing their perspectives

For peer review only - http://bmjopen.bmj.com/site/about/guidelines.xhtml

listening to others and sharing knowledge and experiences students concluded regarding patient care. It can be illustrated by the following quotation:

"To do it [Scenario] *interprofessional adds another dimension… that is very good… instead of just solving the case with my study colleagues" … "... everyone has shared their knowledge... I have listened...and reflected on how to act in a similar situation in my context"* (OT student, activity #3).

Nurse student discussed that both CRM and SBAR also supported interprofessional reasoning when discussing the care for the patient during the different transitions:

"I have little experience of the emergency care… the discussions have been good to prepare myself... knowledge about whom I should turn to, who has the knowledge, what information I should pass on, what will they want to know" (nurse student, activity #4).

The facilitators addressed the gap in the discussion when not all healthcare professionals were represented in the group of students who participated in the current study. In such cases, the facilitators took an active part in the discussion using their own clinical experiences to secure the authenticity and acceptability of the newly developed IPL activity. They contributed with their perspectives and knowledge to facilitate IPL and collaborative learning, including their learning from both the students and the other participating facilitators. However, the facilitators' participation was an act of balancing as the activity was developed for the students and they needed the space to discuss and solve the case in collaboration with the other students.

Discussion

In our study, we explored IPL through the lens of collaborative reasoning using an authentic scenario presented in a digital IPL activity. By examining students, facilitators,

For peer review only - http://bmjopen.bmj.com/site/about/guidelines.xhtml

Begränsad delning

and observers' experiences of the activity, we have shed light on the acceptability, feasibility, and practical implications of incorporating a digital IPL activity for undergraduate students from different healthcare facilities, particularly in the context of critically ill patient handovers between PHC centre and the ambulance services. The findings show that digital IPL activity in addition to IPL also has a positive impact on students' knowledge of and understanding of how to handle an acute care situation and of the value of collaborative reasoning. The scenario used in the IPL activity broadened the students´ perspective on the chain of care and enhanced discussions and knowledge of various professions' roles and responsibilities. The use of a digital format of an IPL activity allowed learning to overcome geographic constraints and underscored the significance of interprofessional problem-solving within an acute care situation for the students. The findings also showed that when the activity was considered authentic, this enabled the students to communicate and share information and enabled an interprofessional analysis. Therefore, it is reasonable to assume that the developed IPL activity supported students' learning through an interactive group process consisting of collaborative analysis and joint critical reflections, factors that are also described by Reeves et.al. [5] to support IPL. However, there is a need to balance the different professions in their interactions and for the students to maintain their communication while facilitating learning and supporting interprofessional discussions [2, 7, 11]. With all the different healthcare professionals represented in the current IPL activity, the scenario covered different perspectives to achieve an interprofessional perspective on patient care through the entire care process. However, the different group compositions that could not be predicted before each IPL activity clarified the need for a scenario that could be used regardless of which professions participated in the IPL activity. Overall, it seemed that the digital IPL activity with a scenario, including a patient with an acute illness, was feasible and relevant for all healthcare

For peer review only - http://bmjopen.bmj.com/site/about/guidelines.xhtml

professionals as they should be able to initiate care in an acute situation. However, out of this study, we do not know the effects of the uneven distribution among different health professions in the IPL activity; facilitators became more active and participated in the discussions by using their clinical profession when necessary. According to both facilitators and students, the facilitators had a direct impact on how the IPL activity was carried out. Students expressed a desire for more guidance and support from the facilitators, while facilitators recognized the importance of incorporating various professions' knowledge and perspectives into the clinical reasoning process for discussing optimal patient care. During the IPL activity, the facilitators employed different strategies to enhance the IPL. These strategies included encouraging active student participation, contributing additional knowledge to the scenario and discussions, asking questions to promote reflection, and guiding students in summarizing the discussions and patient findings using e.g., SBAR. The supervisors used facilitation strategies aligned with strategies identified in the Evans et.al. review [34]. The facilitators in the study did not represent all professionals of the students participating in all IPL activities, as suggested in previous literature [35]. In our study, when the facilitators did not represent all students' disciplines in the activity, they met before, after, and during breaks, and sent text messages during each activity to ensure that perspectives from all healthcare professionals were considered and discussed during the scenario. In addition, the meetings aimed to adjust the facilitators' different roles and strategies as suggested in a previous study [36]. It may be considered important that the facilitator's healthcare professions represent all the various disciplines of students participating in an IPL activity, but it may also be of more importance that the students themselves represent the different disciplines in the discussions. However, further research is needed to clarify whether facilitators need to represent all the various disciplines of students participating in an IPL activity. Finally, to our knowledge, this

For peer review only - http://bmjopen.bmj.com/site/about/guidelines.xhtml

Begränsad delning

study is the first to describe a collaboration between a PHC centre and the ambulance service in developing a digital IPL activity for students. This collaboration can facilitate further cooperation between two different healthcare organizations to enhance safe patient care.

Conclusion

The main findings of this study showed that the digital IPL activity and scenario developed were feasible for enhancing IPL related to acute situations, handover, and patient safety. The positive impact of IPL concerning understanding and knowledge of different professions' perspectives on care and treatment in acute situations, particularly in the ambulance service and PHC context was evident. The findings also underscored the central role of facilitators in promoting collaborative reasoning, emphasizing the need for a balanced and inclusive approach for optimal IPL outcomes. The developed digital IPL activity demonstrated potential sustainability as the digital approach supported overcoming geographical distances for both students and facilitators. By using a scenario involving an authentic case focusing on handovers of a critically ill patient the feasibility and acceptability of using the developed digital activity for IPL were supported. By using digital solutions for the IPL activity, the ambulance service can offer IPL activities in collaboration with other healthcare settings for students doing their clinical rotations in the ambulance service. Finally, to our knowledge, this study is the first to describe a collaboration between a PHC centre and the ambulance service in developing a digital IPL activity for students. This collaboration can facilitate further cooperation between two different healthcare organizations to enhance safe patient care.

Limitations

The study has several limitations. Firstly, the number of participants and completed IPL activities could be considered as low. All students doing their clinical rotation in the PHC centre

For peer review only

or the ambulance service when the activities were conducted were invited by e-mail and/or electronic bulletin board, but we do not know if all eligible students were reached. Secondly, not all professions among the students were representative during IPL activities. This was adjusted for by the facilitators taking an active professional role in the IPL activity, which may have caused a decrease in students' participation in the discussions. However, this was not obvious during the analysis, but we do not know how the facilitators affected the IPL when the students did not represent all healthcare professionals. The third limitation of this study may have been the authors' preconceptions and personal experiences may have influenced the interpretation and understanding of the study. The fact that the authors are female and have extensive clinical experience in the ambulance service or the PHC, and the fifth author is an intensive care nurse and IPL promotor, may introduce biases during the study design, data collection, and analysis. Despite the potential for bias, the authors' preconceptions can also be seen as a strength as they contribute to interpreting and understanding the study findings. Despite these limitations, this is the first study using data from IPL learning activities with students in clinical rotations in both the PHC centre and the ambulance service. Another strength of this study is that for the students it was reasonable to assume that the IPL activity gave the students a possibility for IPL from the perspective of patient safety and patient needs in an acute situation. The third strength of the current study are multiple data sources (interviews, notes, observations) and 31 participants creating new knowledge based on qualitative analysis. Concerning the transferability of the study's findings, as the study is based on IPL, CRM, SBAR, guidelines, and patient safety, considered as generic elements within healthcare services it is reasonable to assume that the findings from this study are transferable to different contexts where interprofessional education and learning on acute situations and patient handovers takes place. Using a digital activity for IPL was considered to facilitate

For peer review only

sustainability, which can enhance transferability and collaboration among different healthcare providers with students doing their clinical rotations in different geographical areas. However quantitative and qualitative evaluation is warranted of IPL learning activities with authentic scenarios in acute transition situations over a longer period of clinical rotations and with a higher number of students.

Abbreviations

CRM	Crew resource management
ECG	Electrocardiography
IPL	Interprofessional learning
OT	Occupational therapy
PHC	Primary Health Care
PHCN	Primary health care nurse
PT	Physiotherapy
SBAR	Situation-Background-Assessment-Recommendation

Declarations

Ethics approval and consent to participate

This study was approved by Etikprövnings myndigheten (The ethics review authority), Sweden (No:2021-00586). Ethical principles by the Declaration of Helsinki were followed [37]. Participants received both oral and written information about the study describing the aim of the study and stating that their participation was voluntary and that they could withdraw at any time without explanation. Informed consent was obtained from all participants in the study.

Consent for publication

Not applicable

For peer review only - http://bmjopen.bmj.com/site/about/guidelines.xhtml

Availability of data and materials

The data analyzed during the current study are not publicly available due to the participants' privacy could be compromised but are available in Swedish from the corresponding author upon reasonable request.

Competing interests

The authors declare no conflicts of interest.

Funding

Karolinska Institutet, Stockholm Sweden (educational funds for employees no: 20200792) and Region Stockholm (ALF project no: 20200223). No person from the founding committee participated in the design of the study, data collection, analysis of data, or in writing the manuscript.

Author contributions

The first, second, and last authors contributed during the study planning. VL, NÅ, and SG collected data and VL initiated data analysis, HC and MT supported the data analysis. MT organized and coordinated learning activities at the Academic primary health care centre. VL drafted the manuscript and all authors contributed, read, and approved the final manuscript.

Acknowledgments

Thank you to all the students who participated in this study, Kristina Björk sharing knowledge on care in the ambulance service, and academic clinical adjuncts Helena Solman, Hege Jahr, and Abdullah Almaasarani.

Authors' information

For peer review only - http://bmjopen.bmj.com/site/about/guidelines.xhtml

For peer review only

Begränsad delning

VL, professor at Umeå University and the ambulance service in the Region of Västerbotten. VL also works as an RN and supervises students in the ambulance service, Samaritan Ambulance. HC, intensive care nurse, senior lecturer, and IPL promotor at Karolinska Institutet. NÅ physiotherapist at Jakobsbergs Rehabilitation Clinic and SG, a specialist nurse in pediatrics at Vällingby Child Health Care Centre, both working in primary health care and are academic clinical adjuncts and facilitators from Academic primary health care centre and MT is an associate professor at Karolinska Institutet and an academic coordinator of on-site training where theoretical knowledge in pedagogy integrates with professional skills within primary healthcare practice for students, research, and continuing education for healthcare providers.

References

1. Suserud BO: **A new profession in the pre-hospital care field--the ambulance nurse**. *Nurs Crit Care* 2005, **10**(6):269-271.
2. Conte H, Wihlborg J, Lindström V: **Developing new possibilities for interprofessional learning- students' experience of learning together in the ambulance service**. *BMC Med Educ* 2022, **22**(1):192.
3. Walker L, Cross M, Barnett T: **Mapping the interprofessional education landscape for students on rural clinical placements: an integrative literature review**. *Rural Remote Health* 2018, **18**(2):4336.
4. Reeves S, Pelone F, Hendry J, Lock N, Marshall J, Pillay L, Wood R: **Using a meta-ethnographic approach to explore the nature of facilitation and teaching approaches employed in interprofessional education**. *Med Teach* 2016, **38**(12):1221-1228.
5. Reeves S, van Soeren M, Macmillan K, Zwarenstein M: **Medicine and nursing: a social contract to improve collaboration and patient-centred care?** *J Interprof Care* 2013, **27**(6):441-442.
6. CAIPE.: **Statement of Purpose CAIPE 2016**. 2016.
7. Conte H, Jirwe M, Scheja M, Hjelmqvist H: **Get it together: Issues that facilitate collaboration in teams of learners in intensive care**. *Med Teach* 2016, **38**(5):491-497.
8. Reeves S, Fletcher S, Barr H, Birch I, Boet S, Davies N, McFadyen A, Rivera J, Kitto S: **A BEME systematic review of the effects of interprofessional education: BEME Guide No. 39**. *Med Teach* 2016, **38**(7):656-668.
9. O'Leary N, Salmon N, Clifford A, O'Donoghue M, Reeves S: **'Bumping along': a qualitative metasynthesis of challenges to interprofessional placements**. 2019, **53**(9):903-915.
10. Oosterom N, Floren LC, Ten Cate O, Westerveld HE: **A review of interprofessional training wards: Enhancing student learning and patient outcomes**. *Med Teach* 2019, **41**(5):547-554.

For peer review only - http://bmjopen.bmj.com/site/about/guidelines.xhtml

11. Croker A, Fisher K, Smith T: **When students from different professions are co-located: the importance of interprofessional rapport for learning to work together**. 2015, **29**(1):41-48.
12. Visser CLF, Ket JCF, Croiset G, Kusurkar RA: **Perceptions of residents, medical and nursing students about Interprofessional education: a systematic review of the quantitative and qualitative literature**. *BMC Med Educ* 2017, **17**(1):77.
13. **Patient Safety** [https://www.who.int/news-room/fact-sheets/detail/patient-safety]
14. McCoy L, Lewis JH, Simon H, Sackett D, Dajani T, Morgan C, Hunt A: **Learning to Speak Up for Patient Safety: Interprofessional Scenarios for Training Future Healthcare Professionals**. *J Med Educ Curric Dev* 2020, **7**:2382120520935469.
15. Young J, Zolio L, Brock T, Harrison J, Hodgkinson M, Kumar A, Morphet J, Kent F: **Interprofessional learning about medication safety**. *Clin Teach* 2021, **18**(6):656-661.
16. Cantero-López N, González-Chordá VM, Valero-Chillerón MJ, Mena-Tudela D, Andreu-Pejó L, Vila-Candel R, Cervera-Gasch Á: **Attitudes of Undergraduate Nursing Students towards Patient Safety: A Quasi-Experimental Study**. *Int J Environ Res Public Health* 2021, **18**(4).
17. Leape L, Berwick D, Clancy C, Conway J, Gluck P, Guest J, Lawrence D, Morath J, O'Leary D, O'Neill P *et al*: **Transforming healthcare: a safety imperative**. *Qual Saf Health Care* 2009, **18**(6):424-428.
18. Mulholland P, Barnett T, Woodroffe J: **A grounded theory of interprofessional learning and paramedic care**. *Journal of Interprofessional Care* 2020, **34**(1):66-75.
19. Wood K, Crouch R, Rowland E, Pope C: **Clinical handovers between prehospital and hospital staff: literature review**. *Emerg Med J* 2015, **32**(7):577-581.
20. **Sentinel Event Data Summary- General Information & 2021 Update** [https://www.jointcommission.org/-/media/tjc/documents/resources/patient-safety-topics/sentinel-event/sentinel-event-general-information-and-2021-update.pdf]
21. Campbell B, Stirling C, Cummings E: **Continuity matters: Examining the 'information gap' in transfer from Residential Aged Care, ambulance to emergency triage in southern Tasmania**. *Int Emerg Nurs* 2017, **32**:9-14.
22. Mulholland P, Barnett T, Spencer J: **Interprofessional learning and rural paramedic care**. *Rural Remote Health* 2014, **14**(3):2821.
23. Mulholland P, O'Meara P, Walker J, Stirling C, Tourle V: **Multidisciplinary Practice in Action: The Rural Paramedic – It's Not Only Lights and Sirens**. *Australasian Journal of Paramedicine* 2009, **7**(2).
24. Bowen DJ, Kreuter M, Spring B, Cofta-Woerpel L, Linnan L, Weiner D, Bakken S, Kaplan CP, Squiers L, Fabrizio C *et al*: **How we design feasibility studies**. *Am J Prev Med* 2009, **36**(5):452-457.
25. Tong A, Sainsbury P, Craig J: **Consolidated criteria for reporting qualitative research (COREQ): a 32-item checklist for interviews and focus groups**. *Int J Qual Health Care* 2007, **19**(6):349-357.
26. Lindström V, Bohm K, Kurland L: **Prehospital care in Sweden**. *Notfall + Rettungsmedizin* 2015, **18**(2):107-109.
27. Soar J, Böttiger BW, Carli P, Couper K, Deakin CD, Djärv T, Lott C, Olasveengen T, Paal P, Pellis T *et al*: **European Resuscitation Council Guidelines 2021: Adult advanced life support**. *Resuscitation* 2021, **161**:115-151.
28. Socialstyrelsen: **National guidelines for cardiac care- Support for control and management (Swe: Nationella riktlinjer för hjärtsjukvård- Stöd för styrning och ledning)**. In.: Socialstyrelsen 2018: 94.
29. Levine GN, Bates ER, Blankenship JC, Bailey SR, Bittl JA, Cercek B, Chambers CE, Ellis SG, Guyton RA, Hollenberg SM *et al*: **2015 ACC/AHA/SCAI Focused Update on Primary Percutaneous Coronary Intervention for Patients With ST-Elevation Myocardial Infarction: An Update of the 2011 ACCF/AHA/SCAI Guideline for Percutaneous Coronary Intervention and the 2013 ACCF/AHA Guideline for the Management of ST-Elevation Myocardial Infarction**. *J Am Coll Cardiol* 2016, **67**(10):1235-1250.

For peer review only - http://bmjopen.bmj.com/site/about/guidelines.xhtml

Begränsad delning

30. **Om personcentrerad vård** [https://www.gu.se/gpcc/om-personcentrerad-vard]
31. Randmaa M, Mårtensson G, Leo Swenne C, Engström M: **SBAR improves communication and safety climate and decreases incident reports due to communication errors in an anaesthetic clinic: a prospective intervention study**. *BMJ Open* 2014, **4**(1):e004268.
32. Weller J, Boyd M, Cumin D: **Teams, tribes and patient safety: overcoming barriers to effective teamwork in healthcare**. *Postgrad Med J* 2014, **90**(1061):149-154.
33. Kiger ME, Varpio L: **Thematic analysis of qualitative data: AMEE Guide No. 131**. *Medical Teacher* 2020, **42**(8):846-854.
34. Evans SM, Ward C, Reeves S: **Online interprofessional education facilitation: A scoping review**. *Medical Teacher* 2019, **41**(2):215-222.
35. Burgess A, Kalman E, Haq I, Leaver A, Roberts C, Bleasel J: **Interprofessional team-based learning (TBL): how do students engage?** 2020, **20**(1):118.
36. Ruiz MG, Ezer H, Purden M: **Exploring the nature of facilitating interprofessional learning: findings from an exploratory study**. *J Interprof Care* 2013, **27**(6):489-495.
37. **World Medical Association-WMA Declaration of Helsinki – Ethical principles for medical research involving human subjects**

For peer review only

For peer review only - http://bmjopen.bmj.com/site/about/guidelines.xhtml

Standards for Reporting Qualitative Research (SRQR)*

http://www.equator-network.org/reporting-guidelines/srqr/

Title and abstract

	Page/line no(s).
**Title** - Concise description of the nature and topic of the study Identifying the study as qualitative or indicating the approach (e.g., ethnography, grounded theory) or data collection methods (e.g., interview, focus group) is recommended	p.1
**Abstract** - Summary of key elements of the study using the abstract format of the intended publication; typically includes background, purpose, methods, results, and conclusions	p.2

Introduction

**Problem formulation** - Description and significance of the problem/phenomenon studied; review of relevant theory and empirical work; problem statement	p.3-7
**Purpose or research question** - Purpose of the study and specific objectives or questions	p. 4

Methods

**Qualitative approach and research paradigm** - Qualitative approach (e.g., ethnography, grounded theory, case study, phenomenology, narrative research) and guiding theory if appropriate; identifying the research paradigm (e.g., postpositivist, constructivist/ interpretivist) is also recommended; rationale**	p.6
**Researcher characteristics and reflexivity** - Researchers' characteristics that may influence the research, including personal attributes, qualifications/experience, relationship with participants, assumptions, and/or presuppositions; potential or actual interaction between researchers' characteristics and the research questions, approach, methods, results, and/or transferability	p.9,21
**Context** - Setting/site and salient contextual factors; rationale**	p.6-7
**Sampling strategy** - How and why research participants, documents, or events were selected; criteria for deciding when no further sampling was necessary (e.g., sampling saturation); rationale**	p.9
**Ethical issues pertaining to human subjects** - Documentation of approval by an appropriate ethics review board and participant consent, or explanation for lack thereof; other confidentiality and data security issues	p. 6, 20
**Data collection methods** - Types of data collected; details of data collection procedures including (as appropriate) start and stop dates of data collection and analysis, iterative process, triangulation of sources/methods, and modification of procedures in response to evolving study findings; rationale**	p.9

For peer review only - http://bmjopen.bmj.com/site/about/guidelines.xhtml

Begränsad delning

**Data collection instruments and technologies** - Description of instruments (e.g., interview guides, questionnaires) and devices (e.g., audio recorders) used for data collection; if/how the instrument(s) changed over the course of the study	p.9
**Units of study** - Number and relevant characteristics of participants, documents, or events included in the study; level of participation (could be reported in results)	p.9
**Data processing** - Methods for processing data prior to and during analysis, including transcription, data entry, data management and security, verification of data integrity, data coding, and anonymization/de-identification of excerpts	p.10
**Data analysis** - Process by which inferences, themes, etc., were identified and developed, including the researchers involved in data analysis; usually references a specific paradigm or approach; rationale**	p.10
**Techniques to enhance trustworthiness** - Techniques to enhance trustworthiness and credibility of data analysis (e.g., member checking, audit trail, triangulation); rationale**	p.10

Results/findings

**Synthesis and interpretation** - Main findings (e.g., interpretations, inferences, and themes); might include development of a theory or model, or integration with prior research or theory	p.10-15
**Links to empirical data** - Evidence (e.g., quotes, field notes, text excerpts, photographs) to substantiate analytic findings	p.10-15

Discussion

**Integration with prior work, implications, transferability, and contribution(s) to the field -** Short summary of main findings; explanation of how findings and conclusions connect to, support, elaborate on, or challenge conclusions of earlier scholarship; discussion of scope of application/generalizability; identification of unique contribution(s) to scholarship in a discipline or field	p.15-18
**Limitations** - Trustworthiness and limitations of findings	p.18

Other

**Conflicts of interest** - Potential sources of influence or perceived influence on study conduct and conclusions; how these were managed	p.20
**Funding** - Sources of funding and other support; role of funders in data collection, interpretation, and reporting	p.21

*The authors created the SRQR by searching the literature to identify guidelines, reporting standards, and critical appraisal criteria for qualitative research; reviewing the reference lists of retrieved sources; and contacting experts to gain feedback. The SRQR aims to improve the transparency of all aspects of qualitative research by providing clear standards for reporting qualitative research.

For peer review only

**The rationale should briefly discuss the justification for choosing that theory, approach, method, or technique rather than other options available, the assumptions and limitations implicit in those choices, and how those choices influence study conclusions and transferability. As appropriate, the rationale for several items might be discussed together.

Reference:

O'Brien BC, Harris IB, Beckman TJ, Reed DA, Cook DA. **Standards for reporting qualitative research: a synthesis of recommendations.** *Academic Medicine*, Vol. 89, No. 9 / Sept 2014 DOI: 10.1097/ACM.0000000000000388

For peer review only

COREQ (COnsolidated criteria for REporting Qualitative research) Checklist

A checklist of items that should be included in reports of qualitative research. You must report the page number in your manuscript where you consider each of the items listed in this checklist. If you have not included this information, either revise your manuscript accordingly before submitting or note N/A.

Topic	Item No.	Guide Questions/Description	Reported on Page No.
**Domain 1: Research team and reflexivity**			
Personal characteristics			
Interviewer/facilitator	1	Which author/s conducted the interview or focus group?	
Credentials	2	What were the researcher's credentials? E.g. PhD, MD	
Occupation	3	What was their occupation at the time of the study?	
Gender	4	Was the researcher male or female?	
Experience and training	5	What experience or training did the researcher have?	
Relationship with participants			
Relationship established	6	Was a relationship established prior to study commencement?	
Participant knowledge of the interviewer	7	What did the participants know about the researcher? e.g. personal goals, reasons for doing the research	
Interviewer characteristics	8	What characteristics were reported about the inter viewer/facilitator? e.g. Bias, assumptions, reasons and interests in the research topic	
**Domain 2: Study design**			
Theoretical framework			
Methodological orientation and Theory	9	What methodological orientation was stated to underpin the study? e.g. grounded theory, discourse analysis, ethnography, phenomenology, content analysis	
Participant selection			
Sampling	10	How were participants selected? e.g. purposive, convenience, consecutive, snowball	
Method of approach	11	How were participants approached? e.g. face-to-face, telephone, mail, email	
Sample size	12	How many participants were in the study?	
Non-participation	13	How many people refused to participate or dropped out? Reasons?	
Setting			
Setting of data collection	14	Where was the data collected? e.g. home, clinic, workplace	
Presence of non-participants	15	Was anyone else present besides the participants and researchers?	
Description of sample	16	What are the important characteristics of the sample? e.g. demographic data, date	
Data collection			
Interview guide	17	Were questions, prompts, guides provided by the authors? Was it pilot tested?	
Repeat interviews	18	Were repeat inter views carried out? If yes, how many?	
Audio/visual recording	19	Did the research use audio or visual recording to collect the data?	
Field notes	20	Were field notes made during and/or after the inter view or focus group?	
Duration	21	What was the duration of the inter views or focus group?	
Data saturation	22	Was data saturation discussed?	
Transcripts returned	23	Were transcripts returned to participants for comment and/or	

For peer review only

Topic	Item No.	Guide Questions/Description	Reported on Page No.
		correction?	
**Domain 3: analysis and findings**			
Data analysis			
Number of data coders	24	How many data coders coded the data?	
Description of the coding tree	25	Did authors provide a description of the coding tree?	
Derivation of themes	26	Were themes identified in advance or derived from the data?	
Software	27	What software, if applicable, was used to manage the data?	
Participant checking	28	Did participants provide feedback on the findings?	
Reporting			
Quotations presented	29	Were participant quotations presented to illustrate the themes/findings? Was each quotation identified? e.g. participant number	
Data and findings consistent	30	Was there consistency between the data presented and the findings?	
Clarity of major themes	31	Were major themes clearly presented in the findings?	
Clarity of minor themes	32	Is there a description of diverse cases or discussion of minor themes?	

Developed from: Tong A, Sainsbury P, Craig J. Consolidated criteria for reporting qualitative research (COREQ): a 32-item checklist for interviews and focus groups. *International Journal for Quality in Health Care*. 2007. Volume 19, Number 6: pp. 349 – 357

Once you have completed this checklist, please save a copy and upload it as part of your submission. DO NOT include this checklist as part of the main manuscript document. It must be uploaded as a separate file.

For peer review only

Facilitating Interprofessional learning: Experiences of using a digital activity for training handover of critically ill patients between a Primary Health Care Centre and Ambulance Services – a qualitative study

Journal:	*BMJ Open*
Manuscript ID	bmjopen-2023-083585.R2
Article Type:	Original research
Date Submitted by the Author:	03-Jun-2024

Complete List of Authors:	Helen, Conte; Karolinska Institutet, Department of Neurobiology, Care Sciences and Society, Division of Nursing<br>Taloyan, Marina; Karolinska Institute, Department of Neurobiology, Care Sciences and Society, Division of Family Medicine and Primary Care; Region Stockholm, Academic primary health care center<br>Ninni, Åkesson; Region Stockholm , Rehab North West<br>Guldbrand, Sofie; Region Stockholm, Child Health Care Centre<br>Lindström, Veronica; Umeå University, Department of Nursing and the Ambulance Service, Västerbotten; Sophiahemmet Hogskola, Department of Health Promotion Science

**Primary Subject Heading**:	Medical education and training
Secondary Subject Heading:	Emergency medicine, Qualitative research, Nursing, Medical education and training
Keywords:	MEDICAL EDUCATION & TRAINING, ACCIDENT & EMERGENCY MEDICINE, Primary Health Care, Feasibility Studies, QUALITATIVE RESEARCH

SCHOLARONE™
Manuscripts

I, the Submitting Author has the right to grant and does grant on behalf of all authors of the Work (as defined in the below author licence), an exclusive licence and/or a non-exclusive licence for contributions from authors who are: i) UK Crown employees; ii) where BMJ has agreed a CC-BY licence shall apply, and/or iii) in accordance with the terms applicable for US Federal Government officers or employees acting as part of their official duties; on a worldwide, perpetual, irrevocable, royalty-free basis to BMJ Publishing Group Ltd ("BMJ") its licensees and where the relevant Journal is co-owned by BMJ to the co-owners of the Journal, to publish the Work in this journal and any other BMJ products and to exploit all rights, as set out in our licence.

The Submitting Author accepts and understands that any supply made under these terms is made by BMJ to the Submitting Author unless you are acting as an employee on behalf of your employer or a postgraduate student of an affiliated institution which is paying any applicable article publishing charge ("APC") for Open Access articles. Where the Submitting Author wishes to make the Work available on an Open Access basis (and intends to pay the relevant APC), the terms of reuse of such Open Access shall be governed by a Creative Commons licence – details of these licences and which Creative Commons licence will apply to this Work are set out in our licence referred to above.

Other than as permitted in any relevant BMJ Author's Self Archiving Policies, I confirm this Work has not been accepted for publication elsewhere, is not being considered for publication elsewhere and does not duplicate material already published. I confirm all authors consent to publication of this Work and authorise the granting of this licence.

For peer review only

Facilitating Interprofessional learning: Experiences of using a digital activity for training handover of critically ill patients between a Primary Health Care Centre and Ambulance Services – a qualitative study

Authors: Conte Helen[1*] Taloyan Marina[2,3] Åkesson Ninni[4], Guldbrand Sofie[5], Lindström Veronica[6,7]

Affiliations:

[1] Department of Neurobiology, Care Sciences and Society, Section of Nursing, Karolinska Institutet, Stockholm, Sweden

[2]Department of Neurobiology, Care Sciences and Society, Division of Family Medicine and Primary Care, Karolinska Institutet, Stockholm, Sweden

[3] Academic primary health care centre, Region Stockholm, Sweden

[4] Rehab North West, Region Stockholm, Sweden

[5] Child Health Care Centre, Region Stockholm; Sweden

[6]Department of Nursing, section of Ambulance Service, Region Västerbotten, Sweden.

[7] Department for Health Promotion Science, Sophiahemmet University, Stockholm, Sweden.

[*] co-first authorship

Word count: 6844

Corresponding author:
Veronica Lindström
Orcid ID: 0000-0003-1386-3203

Department of Nursing, Umeå University & Department of Ambulance Service, Region Västerbotten, Umeå, Sweden.
e-mail: veronica.lindstrom@umu.se
Telephone: +4670-3428157

For peer review only - http://bmjopen.bmj.com/site/about/guidelines.xhtml

Begränsad delning

Abstract

Objective: To explore students' and facilitators' experiences of using a developed digital activity for interprofessional learning (IPL) focusing on critically ill patient handovers from a Primary Health Care (PHC) centre to the ambulance service. **Design**: A qualitative study design was employed, and the reporting of this study adheres to the COREQ guidelines for qualitative studies. **Setting:** A PHC centre and the ambulance service in Stockholm, Sweden**. Participants:** A total of 31 participants were included in the study: 22 students from five different healthcare professions, seven facilitators, and two observers. **Intervention:** A digital IPL activity was developed to overcome geographical distances, and the scenario included the handover of a critically ill patient from personnel within the PHC centre to the ambulance service personnel for transport to an emergency department. Four digital IPL activities were conducted in 2021. **Results:** The digital IPL activity eliminated the issue of geographical distance for students and facilitators, and it enabled the students to find an interprofessional model for collaboration through reasoning, by communicating and sharing knowledge with the support of a common structure. Participants perceived the digital IPL activity and scenario as authentic, feasible, and facilitated IPL. Using a case with an acute and life-threatening condition was a success factor for students to experience high realism in their IPL on patient safety, handover, care, and treatment. **Conclusion:** The developed digital IPL activity facilitated the students' IPL and demonstrated potential sustainability as the digital approach supported overcoming geographical distances for both students and facilitators. By using a scenario involving an authentic case focusing on handovers of a critically ill patient, IPL, feasibility, and acceptability were supported. However, it is crucial to emphasize that a comprehensive evaluation, both quantitative and qualitative, over an extended period of clinical rotations and involving a

For peer review only

For peer review only - http://bmjopen.bmj.com/site/about/guidelines.xhtml

larger group of students is still warranted to ensure continuous improvement and development.

Keywords: Feasibility**,** Interprofessional learning, Interprofessional education, Patient safety; Digital learning activity; Chain of care; Emergency Medical Service; Primary healthcare care service

Strengths and limitations of this study (five bullet points)

- To our knowledge, this study is the first to collect data from digital IPL learning activities with students in clinical rotations in both the Primary Health Care Centre and the ambulance service.
- Multiple data sources (interviews, notes, observations) and 31 participants creating new knowledge based on qualitative analysis.
- The number of participants and completed IPL activities could be considered low, and we do not know if all eligible students were reached.
- Not all professions were representative among the students during all IPL activities.
- The authors' preconceptions and personal experiences may have influenced the interpretation and understanding of the study.

For peer review only

Introduction

Establishing interprofessional learning (IPL) activities that ensure high-quality education is essential. This is particularly critical in clinical settings where IPL is still in the early stages of development. In the ambulance service, IPL is not yet firmly established. Historically, the ambulance service has been perceived as suboptimal for undergraduate students' clinical rotations, primarily due to the services' previous function of responding to emergency calls

For peer review only - http://bmjopen.bmj.com/site/about/guidelines.xhtml

Begränsad delning

and conveying sick or injured individuals to hospitals. However, as the ambulance service has developed into a recognized discipline of pre-hospital emergency care, delivering advanced medical interventions and care [1], it has been shown that within the ambulance service, students can gain proficiency in nursing, medical, and collaborative skills [2]. As the number of undergraduate nursing students completing clinical rotations in the ambulance service has increased, so have the expectations for activities to be conducted to support the students' IPL. However, different student professions undertaking clinical rotations in the ambulance service are limited. Therefore, in collaboration with other healthcare organizations such as primary health care (PHC) centre, there is a need to develop and systematically evaluate easily accessible IPL activities that are not dependent on the co-location of students. IPL activities have been developed and evaluated over the years, but the setting and context matter [3], and contextual factors influence IPL [3]. Therefore, this study aimed to explore students' and facilitators' experiences using a developed digital activity for IPL focusing on critically ill patient handovers from the PHC centre to the ambulance service.

Background

IPL includes various activities depending on goals and desired outcomes, e.g., short digital seminars or shadowing where the students' learning originates from interactive group processes, including collaborative analysis and joint critical reflection [4]. The students are active participants and learn with and from each other in authentic and patient-centred activities [5]. They negotiate their different experiences to fully understand their role and the roles of other professions [6]. However, finding places, spaces, and time for students from various educational programs to learn together in healthcare can be challenging since education is not always situated in contexts that support IPL [7]. Environments and care activities that naturally could support IPL are situated during clinical rotations where students

For peer review only

For peer review only - http://bmjopen.bmj.com/site/about/guidelines.xhtml

from different educational programs collaborate in patient care [8, 9]. Students acknowledged that experiencing diverse IPL activities during clinical rotations is vital for learning how to work together [10]. Co-location is important but not enough for different professions to learn together [10]. Facilitated learning is part of building interprofessional rapport in groups, and balancing the participation of the different professions in their interactions is vital for learning together [2, 6, 10]. Students suggest that bringing a sense of their profession into the interactions and being inclusive of other professions are two key qualities for IPL [10] and that gaps in role conceptions are a crucial barrier [11] and could cause patient risks.

The World Health Organization (WHO) states that healthcare services should be safe and person-centred [12]. Research has shown that IPL activities can introduce patient safety concepts and experiential practice through interprofessional communication and analysis among students [13, 14]. To improve patient safety in the healthcare setting, training must start with students [15, 16]. Working as a professional in the ambulance service involves collaborating with various healthcare professionals when providing care for patients transported to emergency departments or when responding to incidents in home healthcare settings [17]. There is limited knowledge about how undergraduate students perceive digital IPL activities focusing on handovers of critically ill patients within and between the PHC centre and the ambulance service. The handover between different healthcare settings is a complex process that includes communicating information between professionals responsible for the patient's care at different units or sites [18], and communication failures are one of the leading causes of adverse events, particularly in handover situations [19]. When the handover is compromised, essential information can be altered or lost, transfer of cognitive bias may occur, patients may be exposed to adverse events, and patient safety issues may occur [20]. Expanding IPL activities beyond organizational boundaries to include the handover of patients

For peer review only

For peer review only - http://bmjopen.bmj.com/site/about/guidelines.xhtml

Begränsad delning

between different healthcare organizations could allow students to practice and learn handover and patient safety in a new context. The ambulance service collaborates with PHC and community-based care and receives and hands over patients within these organizations [21, 22]. To our knowledge, the handover of critically ill patients from the PHC centre to the ambulance service has not previously been utilized for IPL.

Methods

Design

A qualitative study design was used to collect and analyse the students' and facilitators' experiences through their reflections on the newly developed digital IPL activity based on an authentic scenario. By examining the experiences of supervisors and students, conclusions can be reached regarding IPL in a sparsely explored IPL context, as well as the acceptability and feasibility of the developed digital IPL activity. Acceptability captures how the intended recipients react to the activity, i.e., is it appropriate, relevant, or sustainable [23], and feasibility captures if the developed digital IPL activity is sustainable for continual use in the setting. The consolidated criteria for reporting qualitative research COREQ guidelines guided the study [24]. The study was approved by the ethics review authority in Sweden (No:2021-00586).

Patient and Public Involvement

No patient and public involvement

Setting

The PHC centre provides clinical rotations for medical-, nursing-, physiotherapy (PT)-, occupational therapy students (OT), and nurses studying at an advanced level to become primary health care nurses (PHCNs). In the PHC centre, the learning environment is characterized by care activities of acute and non-acute visits, short and long patient

For peer review only - http://bmjopen.bmj.com/site/about/guidelines.xhtml

encounters, and the students care for patients with all varieties of complaints. The learning environment varies between care for patients at home and the clinic; some patients are followed up on over time, and some only visit the clinic once. The PHC centre has established IPL activities but has never collaborated with the ambulance service concerning IPL activities for undergraduates. The ambulance service provides regular clinical rotations for nursing students and medical students more irregularly. The ambulance service responds to emergency calls, and the students care for patients with various complaints and acuity. Short patient care encounters characterize the learning environment in the ambulance service, care at patients' homes, at the scene of an injury, and during transports between different healthcare facilities. The team in the ambulance consists of an emergency medical technician with basic life support competence and a registered nurse (RN) with one year of additional training in emergency care [25]. The clinical supervisor for both medical and nursing students in the ambulance service is the RN. Collaboration between PHC centres and the ambulance service primarily revolves around the handover of patients suffering from acute illness and needing to be transported from the PHC centre to an emergency department. With two different healthcare organizations collaborating and a lack of co-location, a prerequisite for conducting the IPL activity was that they were conducted digitally. In addition, using a digital format for the IPL activity increased the possibilities for students to participate and increased environmental sustainability by reducing unnecessary travel.

Developing the interprofessional learning activity and the scenario used

The scenario was developed by facilitators and researchers who were clinicians working in the ambulance service, PHC centre, or intensive care unit. To ensure that students from ambulance services and PHC centre could collaborate and contribute with their various professional knowledge, the content of the scenario included (1) situations where different

For peer review only - http://bmjopen.bmj.com/site/about/guidelines.xhtml

Begränsad delning

healthcare professionals cared for the patient, (2) handovers of patients in acute situations, and (3) patient safety in the context of PHC and the ambulance service. The scenario was built on several sources: (1) evidence-based guidelines [26-28], (2) perspectives of person-centered care [29], (3) the Situation-Background-Assessment-Recommendation (SBAR) communication tool [30], and finally, (4) Crew Resource Management (CRM) [31]. After developing the scenario, short sequences were recorded with a smartphone to illustrate different aspects of the care and handover situations. To reduce costs, the patient was acted by one of the facilitators and the recording was made by a clinical colleague from the ambulance service. In total, approximately six working hours were required for the development, filming, and editing of the scenario and for embedding the material into a PowerPoint presentation. The scenario included critical events in the patient's symptom development when suffering from an acute myocardial infarction leading to a cardiac arrest and, after advanced cardio-pulmonary resuscitation (CPR), return to spontaneous circulation. The scenario also included handover situations and supplementary information (e.g., vital signs and ECG). There was a balance between information and questions related to each healthcare profession to enable the students to learn from each profession during the scenario. Questions about CRM and SBAR were added in connection to each care transition in the scenario. The scenario included three episodes of handovers: 1) physiotherapist – primary health care nurse, 2) primary health care nurse or physician or physiotherapist – dispatcher at the emergency medical communication centre, 3) physiotherapist or primary care nurse or physician – ambulance personnel. The IPL activity was completed within 150 minutes, including a 15-minute break.

Data collection and participants

The study and data collection were done in Stockholm, Sweden, where students did their clinical rotations in the ambulance service or at a PHC centre. The students invited to

For peer review only - http://bmjopen.bmj.com/site/about/guidelines.xhtml

participate and evaluate the developed IPL activity were from three higher education institutions. The digital IPL activities were scheduled when there were eligible students from different educational programs at their clinical rotations in the PHC centre or the ambulance service at the same time. The students were invited to participate in the IPL activity through e-mail and/or electronic bulletin boards and had no previous relationship with the researchers (VL, MT, HC). Some facilitators had prior knowledge about the students at the PHC centre as they also worked as clinical teachers (NÅ, SG), but they were not responsible for the students' grading. Data collection took place on four different occasions, once during the spring of 2021 and three times during the fall of 2021.

A combination of data sources was used to capture the participant's experiences in and on the activity. The students' and facilitators' reflective interplay during the IPL activity and group discussions after the activity were collected (a total of 12 hrs of recording) using the digital platform recording function (image and sound). After each IPL activity, separate group discussions were held with the facilitation team. Data collection from the student and facilitator group discussions was initiated with an open-ended question ("What was your experience of this IPL activity?" versus "What did you think about this activity?"). The facilitator's reflective notes were collected after each IPL activity.

In total, 22 students were consecutively included in the study, and seven facilitators and two observers participated in the IPL activity. The scenario was first piloted in the spring of 2021 with eight students, three interprofessional facilitators, and two observers. The observers in this first scenario took notes on body language, group dynamics, and reflections. The results of the analysis of the pilot led to minor adjustments with the addition of a normal ECG and reminders in the PowerPoint to discuss CMR and SBAR. The pilot was included in the analysis.

For peer review only - http://bmjopen.bmj.com/site/about/guidelines.xhtml

Begränsad delning

After piloting, three additional activities with 14 students from five educational programs (medical, nursing, PT, OT, and PHCN) were performed during the fall of 2021. In each activity, three facilitators participated. The interprofessional team of facilitators consisted of clinical teachers with previous experiences in IPL activities: two physical therapists, one specialist nurse with extensive expertise in the ambulance service, one specializing in pediatrics, one in primary healthcare, and one physician. Participating facilitators in the activities were determined by the opportunity to participate. The recordings were transcribed verbatim, and the reflective notes were electronically transcribed.

Analysis

The qualitative materials were analysed thematically, and the analysis was conducted according to the steps described by Kiger and Varpio [32]. Initially, the transcribed text and field notes from the four IPL activities were read multiple times to gain familiarity with the collected material. After this, codes were identified from the texts, and this step was followed by a search and construction of preliminary themes by sorting the identified codes. The preliminary themes were then refined and labelled. In the final step, the analysis was concluded by writing the results. No distinction was made in the analysis regarding either where the students did their clinical rotations or from which programme. Both medical and nursing students can do their clinical rotations in the ambulance service or the PHC centre. Neither were the quotes from the participants, distinguishing as this study aimed to explore on group levels both students' and facilitators' experiences of using a developed digital activity for IPL, focusing on critically ill patient handovers from a PHC centre to the ambulance service. The analyse process was iterative; a continuous movement between the codes, preliminary themes, and themes was made to preserve the essence of the collected data. Each step in the

For peer review only - http://bmjopen.bmj.com/site/about/guidelines.xhtml

process was initiated by the last author and then reviewed by the co-authors. Any differences in interpretation were resolved through discussion until a consensus was reached.

Results

Three themes describing participants' experiences of IPL, the acceptability, and the feasibility of the developed and used IPL activity, from both the students' and facilitators' perspectives, were constructed through the analysis: 1) *Interprofessional learning through collaborative reasoning in an authentic scenario,* 2) *Sharing a common model by communicating information in a structured way,* and 3) *reaching an understanding through collaborative reasoning*.

Interprofessional learning through collaborative reasoning in an authentic scenario

All participants discussed and evaluated the scenario as authentic and based on the patients' needs during handover in an acute situation. The students perceived the activity as a feasible and beneficial way of learning through analysing the patient's changing problems and needs between and with different professions throughout the whole chain of care. The students experienced d that the IPL activity expanded their understanding since they usually did not get a chance to find out what happened with the patient before or after a care encounter. In addition, the students discussed that the developed scenario supported them in their discussions about the patient´s follow-up care at PHC after an acute illness and the support needed for the patient's family.

"…a strength of today's seminar was that many different aspects of care, including discussions on the follow-up of the patient, became clear..." (nurse student, activity #3)

For peer review only - http://bmjopen.bmj.com/site/about/guidelines.xhtml

Begränsad delning

The students agreed that all healthcare professionals need to know how to handle acute situations, for example, the CPR scenario. To enhance IPL during clinical rotations, students and facilitators also discussed the feasibility and possibilities of developing scenarios for future digital IPL activities by incorporating new film sequences into the scenario, for example, by changing symptom presentation and/or changing the outcome for the patient and thereby having the possibility to include other students in the IPL activity.

"*Think about it, adding another outcome for the patient in the scenario can make it relevant for other students, e.g., dietician or maybe homecare personnel* (nurse facilitator 1, activity #3).

The facilitators, students, and observers emphasized that sometimes, the medical focus took over the discussions between the students and risked reducing the possibilities for IPL. Facilitators explained several reasons for this. The first reason could be that not all healthcare professionals and care aspects were explicitly presented in the scenario and, therefore, were not discussed or analysed. The second reason was associated with the facilitators themselves.

"*….XXXX* [name of facilitator] *needs to take a step back, keep quiet and wait for the students to talk, discuss and analyse all different aspects of the care*…" (nurse facilitator 3, activity #2).

An additional reason contributing to the medical focus was the composition of the group of participants. When the group consisted of more medical students than nursing students, the discussions had more of a medical perspective. In contrast, when there were more OT or PT students in the group, their care perspective was discussed to a greater extent, and the medical issues in the scenario needed to be clarified more. In these situations, the facilitators needed to ensure that all aspects were discussed to ensure IPL and the acceptability of the

For peer review only - http://bmjopen.bmj.com/site/about/guidelines.xhtml

developed scenario. During the scenario, the facilitators created a space for the students to take control, verbalize, and analyse together by listening, confirming, and adding critical questions when needed. The facilitators encouraged the students to explain and describe their professional aspects when caring for the patient. Facilitators experienced initial uncertainty among the students and guided them to communicate by open and de-dramatizing questions such as: *Do you have any experience with something similar*? … *What good reflection do you have, anyone else? Don't think about right or wrong answers; start reasoning, and together we will find the solution"* (PT facilitator 2 & nurse facilitator 3, activity #2,3,4)

Sharing a common model by communicating information in a structured way

The students discussed how the scenario highlighted the importance of interprofessional communication in an acute situation and that all participants shared patient information in a structured way using SBAR. They described that using the same structure contributed to understanding how to communicate and reach a shared and patient-centred mental model.

" *I know what to say, and the other person knows what to expect*" (PT student, activity #2).

The shared information is depending, on the other hand, on who they were communicating with and that participants were willing to listen to each other´s point of view:

" You need to be an active listener... it doesn't matter what I say if they don't listen" (PHCN student, activity #4).

Facilitators, observers, and students commonly experienced using CRM to discuss leadership and professional responsibilities in care and in handover situations supported the students' learning of who is leading and who is responsible for which part of the care action. A

For peer review only - http://bmjopen.bmj.com/site/about/guidelines.xhtml

Begränsad delning

conclusion was that situated leadership and responsibility should be taken by the person with the best knowledge of handling an acute situation regardless of professionally situated leadership.

"The one with the most experience and knowledge needs to take the lead in a cardiac arrest situation… I may not have the best knowledge…" (medical student, activity #2) and a PT student continued in the same opinion: *"How to use everybody in a team when caring for a patient with a time-critical illness is essential*" (PT student, activity #1).

Reaching an understanding through collaborative reasoning

According to students, the developed IPL activity and scenario facilitated both wider understanding and new knowledge through interprofessional collaborative reasoning. They learned from each other about both the chain of care and the roles and responsibilities of other professions while switching between thinking and speaking from their perspective and listening to others in planning care and medical treatment in both the ambulance service and at the PHC centre. One medical student expressed it as follows:

"The patient had been somewhere else before entering the PHC…", "It [the scenario] had a holistic perspective" …even though I know the physiotherapist profession, I did not understand their roles and responsibilities in the chain of care" (medical student activity #2).

The students experienced the benefits of solving problems in an interprofessional team instead of doing it by themselves or with peers, as nurse students (activity #2) describe:

"…reasoning with different professions gave me more knowledge about what everyone can do and did in the care of the patient" (nurse student, activity #2).

For peer review only - http://bmjopen.bmj.com/site/about/guidelines.xhtml

As students described, IPL in digital form added a new dimension and facilitated learning about each other's professions, knowledge, roles, and responsibilities in different contexts without needing to go between different healthcare facilities. By verbalizing their perspectives, listening to others, sharing knowledge and experiences, the students reached interprofessional conclusions regarding patient care. It can be illustrated by the following quotation:

"To do it [Scenario] *interprofessional adds another dimension… that is very good… instead of just solving the case with my study colleagues" … "... everyone has shared their knowledge... I have listened...and reflected on how to act in a similar situation in my context"* (OT student, activity #3).

The Nurse students discussed that both CRM and SBAR also supported interprofessional reasoning when discussing the care for the patient during the different transitions in and between PHC centre and ambulance service:

"I have little experience of the emergency care… the discussions have been good to prepare myself... knowledge about whom I should turn to, who has the knowledge, what information I should pass on, what will they want to know" (nurse student, activity #4).

The facilitators addressed the gap in the discussion when not all healthcare professionals were represented in the group of students who participated in the current study. In such cases, the facilitators took an active part in the discussion using their own clinical experiences to secure the authenticity and acceptability of the newly developed IPL activity. They contributed with their perspectives and knowledge to facilitate IPL and collaborative learning, including their learning from both the students and the other participating facilitators. However, the facilitators' participation was an act of balancing as the activity was developed for the

For peer review only - http://bmjopen.bmj.com/site/about/guidelines.xhtml

Begränsad delning

students, and they needed the space to discuss and solve the case in collaboration with the other students.

Discussion

The study explored IPL through the lens of collaborative reasoning using an authentic scenario presented in a digital IPL activity for students doing their clinical rotations in the ambulance service or at the PHC centre. A rationale for developing a digital IPL activity was the need for IPL activities in the ambulance service. By examining students, facilitators, and observers' experiences of the activity, the study have shed light on the acceptability, feasibility, and practical implications of incorporating a digital IPL activity for undergraduate students from different healthcare facilities, particularly in the context of critically ill patient handovers between PHC centre and the ambulance services. The findings show that digital IPL activity also positively impacts students' knowledge of and understanding of how to communicate by sharing information in a structured way, how to handle an acute care situation, and the value of collaborative reasoning. The scenario used in the IPL activity broadened all the students´ perspectives on the chain of care and enhanced discussions and knowledge of various professions' roles and responsibilities. The use of a digital format of an IPL activity enabled learning across multiple geographical areas, which was necessary for the ambulance service, and in addition, underscored the importance of interprofessional problem-solving in handovers of patients within an acute care situation between different healthcare organizations. The findings also showed that when the activity was considered authentic, the students were able to communicate and share information, which enabled an interprofessional analysis. Therefore, it is reasonable to assume that the developed IPL activity supported students' learning through an interactive group process consisting of collaborative analysis and joint critical reflections, factors that are also described by Reeves et al. [4] to

For peer review only - http://bmjopen.bmj.com/site/about/guidelines.xhtml

support IPL. However, there is a need to balance the different professions in their interactions and for the students to maintain communication while facilitating learning and supporting interprofessional discussions [2, 6, 10]. With all the different healthcare professionals represented in the current IPL activity, the scenario covered different perspectives to achieve an interprofessional perspective on patient care through the entire care process. However, the different group compositions that could not be predicted before each IPL activity clarified the need for a scenario that could be used regardless of which professions participated in the IPL activity. Overall, it seemed that the digital IPL activity with a scenario including a patient with an acute illness was feasible and relevant for all healthcare professionals as they should be able to initiate care in an acute situation. However, out of this study, we do not know the effects of the uneven distribution among different health professions in the IPL activity; facilitators became more active and participated in the discussions by using their clinical profession when necessary. According to both facilitators and students, the facilitators directly impacted how the IPL activity was carried out. Students expressed a desire for more guidance and support from the facilitators, while facilitators recognized the importance of incorporating various professions' knowledge and perspectives into the clinical reasoning process for discussing optimal patient care. During the IPL activity, the facilitators employed different strategies to enhance the IPL. These strategies included encouraging active student participation, contributing additional knowledge to the scenario and discussions, asking questions to promote reflection, and guiding students to summarize the discussions and patient findings using, e.g., SBAR. The supervisors used facilitation strategies aligned with strategies identified in the Evans et al. review [33]. The facilitators in the study did not represent all professionals of the students participating in all IPL activities, as suggested in previous literature [34]. In our study, when the facilitators did not represent all students'

For peer review only - http://bmjopen.bmj.com/site/about/guidelines.xhtml

disciplines in the activity, they met before, after, and during breaks and sent text messages during each activity to ensure that perspectives from all healthcare professionals were considered and discussed during the scenario. In addition, the meetings aimed to adjust the facilitators' different roles and strategies, as suggested in a previous study [35]. It may be considered important that the facilitator's healthcare professions represent all the various disciplines of students participating in an IPL activity, but it may also be of more importance that the students themselves represent the different disciplines in the discussions as described by Oosterom et al.[9] However, further research is needed to clarify whether facilitators must represent all the various disciplines of students participating in an IPL activity. Regardless, the ambulance service needs to collaborate with other healthcare organizations to be able to conduct IPL activities because the number of different professions within the organization is limited. Finally, to our knowledge, this study is the first to describe a collaboration between a PHC centre and the ambulance service in developing a digital IPL activity for students. This collaboration can facilitate cooperation between two healthcare organizations to enhance safe patient care.

Conclusion

The main findings of this study showed that the digital IPL activity and scenario developed were feasible for enhancing IPL related to acute situations, handover, and patient safety in the context of PHC and the ambulance service. The positive impact of IPL concerning understanding and knowledge of different professions' perspectives on care and treatment in acute situations, particularly in the ambulance service and PHC context, was evident. The findings also underscored the central role of facilitators in promoting collaborative reasoning, emphasizing the need for a balanced and inclusive approach for optimal IPL outcomes. The developed digital IPL activity demonstrated potential sustainability and a necessity for the

For peer review only - http://bmjopen.bmj.com/site/about/guidelines.xhtml

ambulance service as the digital approach supported overcoming geographical distances for both students and facilitators. By using a scenario involving an authentic case focusing on handovers of a critically ill patient, the feasibility and acceptability of using the developed digital activity for IPL were supported. By using digital solutions for the IPL activity, the ambulance service can offer IPL activities in collaboration with other healthcare settings for students doing their clinical rotations in the ambulance service. Finally, to our knowledge, this study is the first to describe a collaboration between a PHC centre and the ambulance service in developing a digital IPL activity for students. This collaboration can facilitate cooperation between two healthcare organizations to enhance safe patient care.

Limitations

The study has several limitations. Firstly, the number of participants and completed IPL activities could be considered low. All students doing their clinical rotation in the PHC centre or the ambulance service when the activities were conducted were invited by e-mail and/or electronic bulletin board, but we do not know if all eligible students were reached. Secondly, not all professions among the students were representative during IPL activities. This was adjusted for by the facilitators taking an active professional role in the IPL activity, which may have caused a decrease in students' participation in the discussions. However, this was not obvious during the analysis, but we do not know how the facilitators affected the IPL when the students did not represent all healthcare professionals. The third limitation of this study may have been the authors' preconceptions and personal experiences, which may have influenced the interpretation and understanding of the study. The fact that the authors are female have extensive clinical experience in the ambulance service or the PHC, and the fifth author is an intensive care nurse and IPL promotor may introduce biases during the study design, data collection, and analysis. Despite the potential for bias, the authors'

For peer review only - http://bmjopen.bmj.com/site/about/guidelines.xhtml

Begränsad delning

preconceptions can also be a strength as they contribute to interpreting and understanding the study findings. Despite these limitations, this is the first study using data from IPL learning activities with students in clinical rotations in both the PHC centre and the ambulance service. Another strength of this study is that it was reasonable to assume that the IPL activity gave the students a possibility for IPL from the perspective of patient safety and patient needs in an acute situation. The third strength of the current study are multiple data sources (interviews, notes, observations) and 31 participants creating new knowledge based on qualitative analysis. Concerning the transferability of the study's findings, as the study is based on IPL, CRM, SBAR, guidelines, and patient safety, considered as generic elements within healthcare services, it is reasonable to assume that the findings from this study are transferable to different contexts where interprofessional education and learning on acute situations and patient handovers take place. Using a digital activity for IPL was considered to facilitate sustainability, which can enhance transferability and collaboration among different healthcare providers with students doing their clinical rotations in different geographical areas. However, quantitative and qualitative evaluation is warranted for IPL learning activities with authentic scenarios in acute transition situations over a longer period of clinical rotations and with a higher number of students.

Abbreviations

CRM	Crew resource management
ECG	Electrocardiography
IPL	Interprofessional learning
OT	Occupational therapy
PHC	Primary Health Care
PHCN	Primary health care nurse
PT	Physiotherapy

For peer review only - http://bmjopen.bmj.com/site/about/guidelines.xhtml

SBAR Situation-Background-Assessment-Recommendation

Declarations

Ethics approval and consent to participate

This study was approved by Etikprövnings myndigheten (the ethics review authority), Sweden (No:2021-00586). Ethical principles outlined in the Declaration of Helsinki were followed [37]. Participants received both oral and written information about the study describing its aim and stating that their participation was voluntary and that they could withdraw at any time without explanation. Informed consent was obtained from all participants in the study.

Consent for publication

Not applicable

Availability of data and materials

The data analyzed during the current study are not publicly available because the participants' privacy could be compromised, but they are available in Swedish from the corresponding author upon reasonable request.

Competing interests

The authors declare no conflicts of interest.

Funding

For peer review only

Karolinska Institutet, Stockholm, Sweden (educational funds for employees no: 20200792), and Region Stockholm (ALF project no: 20200223). No person from the founding committee participated in the study's design, data collection, analysis, or writing of the manuscript.

Author contributions

The first, second, and last authors contributed during the study planning. HC and MT have a co-first authorship. VL, NÅ, and SG collected data and VL initiated data analysis, HC and MT supported the data analysis. MT organized and coordinated learning activities at the Academic primary health care centre. VL drafted the manuscript and all authors contributed, read, and approved the final manuscript.

Acknowledgments

Thank you to all the students who participated in this study, Kristina Björk sharing knowledge on care in the ambulance service, and academic clinical adjuncts Helena Solman, Hege Jahr, and Abdullah Almaasarani.

Authors' information

VL, professor at Umeå University and the ambulance service in the Region of Västerbotten. VL also works as an RN and supervises students in the ambulance service, Samaritan Ambulance. HC, intensive care nurse, senior lecturer, and IPL promotor at Karolinska Institutet. NÅ physiotherapist at Jakobsbergs Rehabilitation Clinic and SG, a specialist nurse in pediatrics at Vällingby Child Health Care Centre, both working in primary health care and are academic clinical adjuncts and facilitators from Academic primary health care centre and MT is an associate professor at Karolinska Institutet and an academic coordinator of on-site training where theoretical knowledge in pedagogy integrates with professional skills within

For peer review only - http://bmjopen.bmj.com/site/about/guidelines.xhtml

primary healthcare practice for students, research, and continuing education for healthcare providers.

References

1. Suserud BO: **A new profession in the pre-hospital care field--the ambulance nurse**. *Nurs Crit Care* 2005, **10**(6):269-271.
2. Conte H, Wihlborg J, Lindström V: **Developing new possibilities for interprofessional learning- students' experience of learning together in the ambulance service**. *BMC Med Educ* 2022, **22**(1):192.
3. Reeves S, Pelone F, Hendry J, Lock N, Marshall J, Pillay L, Wood R: **Using a meta-ethnographic approach to explore the nature of facilitation and teaching approaches employed in interprofessional education**. *Med Teach* 2016, **38**(12):1221-1228.
4. Reeves S, van Soeren M, Macmillan K, Zwarenstein M: **Medicine and nursing: a social contract to improve collaboration and patient-centred care?** *J Interprof Care* 2013, **27**(6):441-442.
5. CAIPE.: **Statement of Purpose CAIPE 2016**. 2016.
6. Conte H, Jirwe M, Scheja M, Hjelmqvist H: **Get it together: Issues that facilitate collaboration in teams of learners in intensive care**. *Med Teach* 2016, **38**(5):491-497.
7. Reeves S, Fletcher S, Barr H, Birch I, Boet S, Davies N, McFadyen A, Rivera J, Kitto S: **A BEME systematic review of the effects of interprofessional education: BEME Guide No. 39**. *Med Teach* 2016, **38**(7):656-668.
8. O'Leary N, Salmon N, Clifford A, O'Donoghue M, Reeves S: **'Bumping along': a qualitative metasynthesis of challenges to interprofessional placements**. 2019, **53**(9):903-915.
9. Oosterom N, Floren LC, Ten Cate O, Westerveld HE: **A review of interprofessional training wards: Enhancing student learning and patient outcomes**. *Med Teach* 2019, **41**(5):547-554.
10. Croker A, Fisher K, Smith T: **When students from different professions are co-located: the importance of interprofessional rapport for learning to work together**. 2015, **29**(1):41-48.
11. Visser CLF, Ket JCF, Croiset G, Kusurkar RA: **Perceptions of residents, medical and nursing students about Interprofessional education: a systematic review of the quantitative and qualitative literature**. *BMC Med Educ* 2017, **17**(1):77.
12. **Patient Safety** [https://www.who.int/news-room/fact-sheets/detail/patient-safety]
13. McCoy L, Lewis JH, Simon H, Sackett D, Dajani T, Morgan C, Hunt A: **Learning to Speak Up for Patient Safety: Interprofessional Scenarios for Training Future Healthcare Professionals**. *J Med Educ Curric Dev* 2020, **7**:2382120520935469.
14. Young J, Zolio L, Brock T, Harrison J, Hodgkinson M, Kumar A, Morphet J, Kent F: **Interprofessional learning about medication safety**. *Clin Teach* 2021, **18**(6):656-661.
15. Cantero-López N, González-Chordá VM, Valero-Chillerón MJ, Mena-Tudela D, Andreu-Pejó L, Vila-Candel R, Cervera-Gasch Á: **Attitudes of Undergraduate Nursing Students towards Patient Safety: A Quasi-Experimental Study**. *Int J Environ Res Public Health* 2021, **18**(4).
16. Leape L, Berwick D, Clancy C, Conway J, Gluck P, Guest J, Lawrence D, Morath J, O'Leary D, O'Neill P *et al*: **Transforming healthcare: a safety imperative**. *Qual Saf Health Care* 2009, **18**(6):424-428.
17. Mulholland P, Barnett T, Woodroffe J: **A grounded theory of interprofessional learning and paramedic care**. *Journal of Interprofessional Care* 2020, **34**(1):66-75.
18. Wood K, Crouch R, Rowland E, Pope C: **Clinical handovers between prehospital and hospital staff: literature review**. *Emerg Med J* 2015, **32**(7):577-581.

For peer review only - http://bmjopen.bmj.com/site/about/guidelines.xhtml

Begränsad delning

19. **Sentinel Event Data Summary- General Information & 2021 Update** [https://www.jointcommission.org/-/media/tjc/documents/resources/patient-safety-topics/sentinel-event/sentinel-event-general-information-and-2021-update.pdf]
20. Campbell B, Stirling C, Cummings E: **Continuity matters: Examining the 'information gap' in transfer from Residential Aged Care, ambulance to emergency triage in southern Tasmania**. *Int Emerg Nurs* 2017, **32**:9-14.
21. Mulholland P, Barnett T, Spencer J: **Interprofessional learning and rural paramedic care**. *Rural Remote Health* 2014, **14**(3):2821.
22. Mulholland P, O'Meara P, Walker J, Stirling C, Tourle V: **Multidisciplinary Practice in Action: The Rural Paramedic – It's Not Only Lights and Sirens**. *Australasian Journal of Paramedicine* 2009, **7**(2).
23. Bowen DJ, Kreuter M, Spring B, Cofta-Woerpel L, Linnan L, Weiner D, Bakken S, Kaplan CP, Squiers L, Fabrizio C *et al*: **How we design feasibility studies**. *Am J Prev Med* 2009, **36**(5):452-457.
24. Tong A, Sainsbury P, Craig J: **Consolidated criteria for reporting qualitative research (COREQ): a 32-item checklist for interviews and focus groups**. *Int J Qual Health Care* 2007, **19**(6):349-357.
25. Lindström V, Bohm K, Kurland L: **Prehospital care in Sweden**. *Notfall + Rettungsmedizin* 2015, **18**(2):107-109.
26. Soar J, Böttiger BW, Carli P, Couper K, Deakin CD, Djärv T, Lott C, Olasveengen T, Paal P, Pellis T *et al*: **European Resuscitation Council Guidelines 2021: Adult advanced life support**. *Resuscitation* 2021, **161**:115-151.
27. Socialstyrelsen: **National guidelines for cardiac care- Support for control and management (Swe: Nationella riktlinjer för hjärtsjukvård- Stöd för styrning och ledning)**. In.: Socialstyrelsen 2018: 94.
28. Levine GN, Bates ER, Blankenship JC, Bailey SR, Bittl JA, Cercek B, Chambers CE, Ellis SG, Guyton RA, Hollenberg SM *et al*: **2015 ACC/AHA/SCAI Focused Update on Primary Percutaneous Coronary Intervention for Patients With ST-Elevation Myocardial Infarction: An Update of the 2011 ACCF/AHA/SCAI Guideline for Percutaneous Coronary Intervention and the 2013 ACCF/AHA Guideline for the Management of ST-Elevation Myocardial Infarction**. *J Am Coll Cardiol* 2016, **67**(10):1235-1250.
29. **Om personcentrerad vård** [https://www.gu.se/gpcc/om-personcentrerad-vard]
30. Randmaa M, Mårtensson G, Leo Swenne C, Engström M: **SBAR improves communication and safety climate and decreases incident reports due to communication errors in an anaesthetic clinic: a prospective intervention study**. *BMJ Open* 2014, **4**(1):e004268.
31. Weller J, Boyd M, Cumin D: **Teams, tribes and patient safety: overcoming barriers to effective teamwork in healthcare**. *Postgrad Med J* 2014, **90**(1061):149-154.
32. Kiger ME, Varpio L: **Thematic analysis of qualitative data: AMEE Guide No. 131**. *Medical Teacher* 2020, **42**(8):846-854.
33. Evans SM, Ward C, Reeves S: **Online interprofessional education facilitation: A scoping review**. *Medical Teacher* 2019, **41**(2):215-222.
34. Burgess A, Kalman E, Haq I, Leaver A, Roberts C, Bleasel J: **Interprofessional team-based learning (TBL): how do students engage?** 2020, **20**(1):118.
35. Ruiz MG, Ezer H, Purden M: **Exploring the nature of facilitating interprofessional learning: findings from an exploratory study**. *J Interprof Care* 2013, **27**(6):489-495.

For peer review only

COREQ (COnsolidated criteria for REporting Qualitative research) Checklist

A checklist of items that should be included in reports of qualitative research. You must report the page number in your manuscript where you consider each of the items listed in this checklist. If you have not included this information, either revise your manuscript accordingly before submitting or note N/A.

Topic	Item No.	Guide Questions/Description	Reported on Page No.
**Domain 1: Research team and reflexivity**			
Personal characteristics			
Interviewer/facilitator	1	Which author/s conducted the interview or focus group?	
Credentials	2	What were the researcher's credentials? E.g. PhD, MD	
Occupation	3	What was their occupation at the time of the study?	
Gender	4	Was the researcher male or female?	
Experience and training	5	What experience or training did the researcher have?	
Relationship with participants			
Relationship established	6	Was a relationship established prior to study commencement?	
Participant knowledge of the interviewer	7	What did the participants know about the researcher? e.g. personal goals, reasons for doing the research	
Interviewer characteristics	8	What characteristics were reported about the inter viewer/facilitator? e.g. Bias, assumptions, reasons and interests in the research topic	
**Domain 2: Study design**			
Theoretical framework			
Methodological orientation and Theory	9	What methodological orientation was stated to underpin the study? e.g. grounded theory, discourse analysis, ethnography, phenomenology, content analysis	
Participant selection			
Sampling	10	How were participants selected? e.g. purposive, convenience, consecutive, snowball	
Method of approach	11	How were participants approached? e.g. face-to-face, telephone, mail, email	
Sample size	12	How many participants were in the study?	
Non-participation	13	How many people refused to participate or dropped out? Reasons?	
Setting			
Setting of data collection	14	Where was the data collected? e.g. home, clinic, workplace	
Presence of non-participants	15	Was anyone else present besides the participants and researchers?	
Description of sample	16	What are the important characteristics of the sample? e.g. demographic data, date	
Data collection			
Interview guide	17	Were questions, prompts, guides provided by the authors? Was it pilot tested?	
Repeat interviews	18	Were repeat inter views carried out? If yes, how many?	
Audio/visual recording	19	Did the research use audio or visual recording to collect the data?	
Field notes	20	Were field notes made during and/or after the inter view or focus group?	
Duration	21	What was the duration of the inter views or focus group?	
Data saturation	22	Was data saturation discussed?	
Transcripts returned	23	Were transcripts returned to participants for comment and/or	

For peer review only

Topic	Item No.	Guide Questions/Description	Reported on Page No.
		correction?	
**Domain 3: analysis and findings**			
Data analysis			
Number of data coders	24	How many data coders coded the data?	
Description of the coding tree	25	Did authors provide a description of the coding tree?	
Derivation of themes	26	Were themes identified in advance or derived from the data?	
Software	27	What software, if applicable, was used to manage the data?	
Participant checking	28	Did participants provide feedback on the findings?	
Reporting			
Quotations presented	29	Were participant quotations presented to illustrate the themes/findings? Was each quotation identified? e.g. participant number	
Data and findings consistent	30	Was there consistency between the data presented and the findings?	
Clarity of major themes	31	Were major themes clearly presented in the findings?	
Clarity of minor themes	32	Is there a description of diverse cases or discussion of minor themes?	

Developed from: Tong A, Sainsbury P, Craig J. Consolidated criteria for reporting qualitative research (COREQ): a 32-item checklist for interviews and focus groups. *International Journal for Quality in Health Care*. 2007. Volume 19, Number 6: pp. 349 – 357

Once you have completed this checklist, please save a copy and upload it as part of your submission. DO NOT include this checklist as part of the main manuscript document. It must be uploaded as a separate file.

For peer review only

Standards for Reporting Qualitative Research (SRQR)*

http://www.equator-network.org/reporting-guidelines/srqr/

Title and abstract

	Page/line no(s).
**Title** - Concise description of the nature and topic of the study Identifying the study as qualitative or indicating the approach (e.g., ethnography, grounded theory) or data collection methods (e.g., interview, focus group) is recommended	p.1
**Abstract** - Summary of key elements of the study using the abstract format of the intended publication; typically includes background, purpose, methods, results, and conclusions	p.2

Introduction

**Problem formulation** - Description and significance of the problem/phenomenon studied; review of relevant theory and empirical work; problem statement	p.3-7
**Purpose or research question** - Purpose of the study and specific objectives or questions	p. 4

Methods

**Qualitative approach and research paradigm** - Qualitative approach (e.g., ethnography, grounded theory, case study, phenomenology, narrative research) and guiding theory if appropriate; identifying the research paradigm (e.g., postpositivist, constructivist/ interpretivist) is also recommended; rationale**	p.6
**Researcher characteristics and reflexivity** - Researchers' characteristics that may influence the research, including personal attributes, qualifications/experience, relationship with participants, assumptions, and/or presuppositions; potential or actual interaction between researchers' characteristics and the research questions, approach, methods, results, and/or transferability	p.9,21
**Context** - Setting/site and salient contextual factors; rationale**	p.6-7
**Sampling strategy** - How and why research participants, documents, or events were selected; criteria for deciding when no further sampling was necessary (e.g., sampling saturation); rationale**	p.9
**Ethical issues pertaining to human subjects** - Documentation of approval by an appropriate ethics review board and participant consent, or explanation for lack thereof; other confidentiality and data security issues	p. 6, 20
**Data collection methods** - Types of data collected; details of data collection procedures including (as appropriate) start and stop dates of data collection and analysis, iterative process, triangulation of sources/methods, and modification of procedures in response to evolving study findings; rationale**	p.9

For peer review only

For peer review only - http://bmjopen.bmj.com/site/about/guidelines.xhtml

Begränsad delning

**Data collection instruments and technologies** - Description of instruments (e.g., interview guides, questionnaires) and devices (e.g., audio recorders) used for data collection; if/how the instrument(s) changed over the course of the study	p.9
**Units of study** - Number and relevant characteristics of participants, documents, or events included in the study; level of participation (could be reported in results)	p.9
**Data processing** - Methods for processing data prior to and during analysis, including transcription, data entry, data management and security, verification of data integrity, data coding, and anonymization/de-identification of excerpts	p.10
**Data analysis** - Process by which inferences, themes, etc., were identified and developed, including the researchers involved in data analysis; usually references a specific paradigm or approach; rationale**	p.10
**Techniques to enhance trustworthiness** - Techniques to enhance trustworthiness and credibility of data analysis (e.g., member checking, audit trail, triangulation); rationale**	p.10

Results/findings

**Synthesis and interpretation** - Main findings (e.g., interpretations, inferences, and themes); might include development of a theory or model, or integration with prior research or theory	p.10-15
**Links to empirical data** - Evidence (e.g., quotes, field notes, text excerpts, photographs) to substantiate analytic findings	p.10-15

Discussion

**Integration with prior work, implications, transferability, and contribution(s) to the field -** Short summary of main findings; explanation of how findings and conclusions connect to, support, elaborate on, or challenge conclusions of earlier scholarship; discussion of scope of application/generalizability; identification of unique contribution(s) to scholarship in a discipline or field	p.15-18
**Limitations** - Trustworthiness and limitations of findings	p.18

Other

**Conflicts of interest** - Potential sources of influence or perceived influence on study conduct and conclusions; how these were managed	p.20
**Funding** - Sources of funding and other support; role of funders in data collection, interpretation, and reporting	p.21

*The authors created the SRQR by searching the literature to identify guidelines, reporting standards, and critical appraisal criteria for qualitative research; reviewing the reference lists of retrieved sources; and contacting experts to gain feedback. The SRQR aims to improve the transparency of all aspects of qualitative research by providing clear standards for reporting qualitative research.

For peer review only - http://bmjopen.bmj.com/site/about/guidelines.xhtml

**The rationale should briefly discuss the justification for choosing that theory, approach, method, or technique rather than other options available, the assumptions and limitations implicit in those choices, and how those choices influence study conclusions and transferability. As appropriate, the rationale for several items might be discussed together.

Reference:

O'Brien BC, Harris IB, Beckman TJ, Reed DA, Cook DA. **Standards for reporting qualitative research: a synthesis of recommendations.** *Academic Medicine*, Vol. 89, No. 9 / Sept 2014 DOI: 10.1097/ACM.0000000000000388

For peer review only
